# Engineered CCR2 Cell Membrane-Wrapped Cepharanthine Liposomes for Potential Targeted Attenuation of Acute Lung Injury

**DOI:** 10.3390/cells15030292

**Published:** 2026-02-04

**Authors:** Yifan Qing, Wenbo Zhao, Liangliang Xue, Yu Luo, Yuhao Gao, Xiang Sun, Fan Li, Linxuan Dai, Jing Mo, Guoqing Xu, Zenghao Bi, Suleixin Yang, Woo Tiam Hee, Jie Li, Liang Leng

**Affiliations:** 1 School of Basic Medical Sciences, Chengdu University of Traditional Chinese Medicine, Chengdu 611137, China; 2 Institute of Herbgenomics, Chengdu University of Traditional Chinese Medicine, Chengdu 611137, China; 3 College of Pharmacy, Chengdu University of Traditional Chinese Medicine, Chengdu 611137, China; 4 School of Medical and Life Sciences, Chengdu University of Traditional Chinese Medicine, Chengdu 611137, China; 5 Innovative Institute of Chinese Medicine and Pharmacy, Chengdu University of Traditional Chinese Medicine, Chengdu 611137, China; 6 M. Kandiah Faculty of Medicine and Health Sciences, University Tunku Abdul Rahman, Kajang 43000, Malaysia

**Keywords:** acute lung injury, engineered CCR2 cell membrane, cepharanthine, liposome, potential targeted strategy, anti-inflammatory

## Abstract

**Highlights:**

**What are the main findings?**

- We developed CEP@LP-M^CCR2^; the original liposome integrates the advantages of cell membranes and lipid materials.
- CEP@LP-M^CCR2^ enables efficient accumulation of CEP in inflamed lungs.
- CEP@LP-M^CCR2^ is equipped with a CCR2-overexpressed surface, enabling it to selectively combine CCL2, which is related to acute lung injury (ALI).
- CEP@LP-M^CCR2^ could reduce the levels of key pro-inflammatory cytokines, suppress M1 macrophage polarization, and upregulate the expression of junctional proteins.
- RNA sequencing indicated that CEP@LP-M^CCR2^ may inactivate the TNF/NF-κB signaling axis.

**What are the implications of the main findings?**

- CEP@LP-M^CCR2^ mitigates the limitations of free CEP, namely, its poor solubility and inferior biocompatibility.
- CEP@LP-M^CCR2^ has the potential to reduce inflammation and recovery barrier function, which could attenuate of ALI.
- CEP@LP-M^CCR2^ offers novel potential targeting.

**Abstract:**

Severe respiratory inflammation or viral infections can lead to acute lung injury (ALI), a disease characterized by diffuse inflammatory injury of the pulmonary epithelium and endothelium. Cepharanthine (CEP) is reported as a promising drug candidate due to its antiviral properties. However, CEP exhibits poor solubility and low bioavailability. Therefore, we developed a novel liposome, named CEP@LP-M^CCR2^, which integrates the advantages of cell membranes and lipid materials, to achieve effective accumulation of CEP in inflamed lungs. It exhibits a 1.73-fold increase in lung accumulation at 24 h in vivo, a 4.56-fold increase in cellular uptake in MLE-12 cells. CEP@LP-M^CCR2^ is equipped with a CCR2-overexpressed surface, enabling it to selectively neutralize elevated levels of CCL2, which is related to ALI, thereby reducing macrophage infiltration, thereby reducing the spread of inflammation, such as a reduction in levels of key pro-inflammatory cytokines (TNF-α, IL-1β, and IL-6). CEP@LP-M^CCR2^ could suppress M1 macrophage polarization, which led to a marked decrease in iNOS and an increase in Arg1. It upregulated the expression of junctional proteins E-cadherin and Occludin, indicating potential recovery of the pulmonary epithelial barrier. RNA sequencing analysis implied the potential of CEP@LP-M^CCR2^ to inactivate the TNF/NF-κB signaling axis.

## 1. Introduction

Acute lung injury (ALI) is a severe pulmonary disorder characterized by the destruction of the alveolar–capillary barrier, leading to alveolar edema and profound gas-exchange impairment [1]. There are numerous pathogenic factors, including severe infection or inflammation [2,3]. For example, infection with coronaviruses, such as SARS-CoV-2 or other homologous variants, triggers a pronounced inflammatory and immune response. This response drives the apoptosis of epithelial and endothelial cells, which in turn induces aberrant activation of macrophages. This cascade ultimately leads to ALI and, in severe cases, death [4,5]. However, current treatment strategies for ALI remain controversial due to their side effects, administration routes, or inadequate targeting.

Cepharanthine (CEP), a secondary metabolite derived from *Stephania* that belongs to benzylisoquinoline alkaloids (BIAs), has garnered attention for its reported anti-inflammatory and inhibition of infection caused by several important human viruses, including SARS-CoV-2. CEP exerts its anti-inflammatory effects by suppressing nuclear-factor-κB (NF-κB) activation [6,7], reducing the production of pro-inflammatory cytokines such as TNF-α [6,7], and inhibiting M1 macrophage polarization [8]. These actions collectively attenuate excessive inflammation. CEP, approved by the Pharmaceuticals and Medical Devices Agency (PMDA) in Japan, is used to treat cancer and inflammation. Against SARS-CoV-2 and its closely related coronaviruses, it is reported that a large-scale screening of 2406 clinically approved drugs by Tong’s team identified CEP is a wide-spectrum inhibitor of pan-β-coronavirus [9]. Chen’s team discovered a group of CEP analogs possess potential broad-spectrum anti-coronavirus activity [10]. Therefore, CEP is a promising candidate drug for treating COVID-19 [9,11]. But CEP has poor solubility and is not easily absorbed by oral administration, resulting in low bioavailability [12], and injectable formulations are rapidly cleared from the body. Consequently, there is a clear need for a superior delivery strategy that can concentrate CEP within the lung. Such a strategy would mitigate effects, permit the lower doses, and attenuate ALI.

Liposomes coated with biologically derived cell membranes have recently attracted attention. Liposomes are defined as small synthetic vesicles composed of one or more lipid bilayers separated by aqueous compartments [13]. Liposomes were first described by Bangham in the mid-1960s; it turned out to be an accidental discovery in which he scattered the phosphatidyl choline molecule in water. For the duration of this he found that the molecule was forming a closed bilayer shape having an aqueous segment that was entrapped by means of a lipid bilayer [14,15]. These liposomes can encapsulate lipid-soluble drugs within the phospholipid bilayers [16]. The biomimetic liposomes of the cell membrane inherit the surface characteristics of the source cell, without complex chemical treatments or traditional synthetic modifications [17]. They can serve as nanoscale decoys, neutralizing the pathological molecules involved in the progression of the disease [18,19,20]; moreover, the different biological functions of the surface proteins on the cell membrane endow nanoparticles with various capabilities, such as immune evasion, long circulation time, and targeted delivery [21]. Diverse cell membranes are exploited in biomimetic systems, including red blood cells [22], platelets [23], cancer cells [24], and macrophages [25]. However, these native cell membranes have several proteins, which may limit targeting specificity and be involved in interacting with the host environment, affecting biodistribution and toxicity profile [26]. In contrast, HEK293T cells boast rapid mitotic and remarkable transfection capabilities for stable target proteins [27,28], making their membranes applicable to biomimetic nanoparticles. Consequently, the combination of engineered HEK293T cells that overexpress target proteins on their surface with liposomes constitutes a viable strategy for the targeted treatment of inflammatory diseases.

The lesion area of ALI can produce and release a variety of pro-inflammatory cytokines, chemokines. In ALI patients and animal models, upregulation of CCL2 expression in pulmonary epithelial cells recruits CCR2-expressing cells (such as monocytes) to exacerbate the inflammatory response. Therefore, CCR2-expressing engineered cell membranes were prepared as biomimetic nanoparticles, which can not only achieve concealment by virtue of the natural biological camouflage properties of the cell membrane but also possess potential targeting capability toward inflamed lung tissues. Therefore, CCR2-expressing-cell-membrane-coated CEP liposomes (CEP@LP) enable potential targeting of ALI, and this may be an effective novel strategy.

**Scheme 1:**
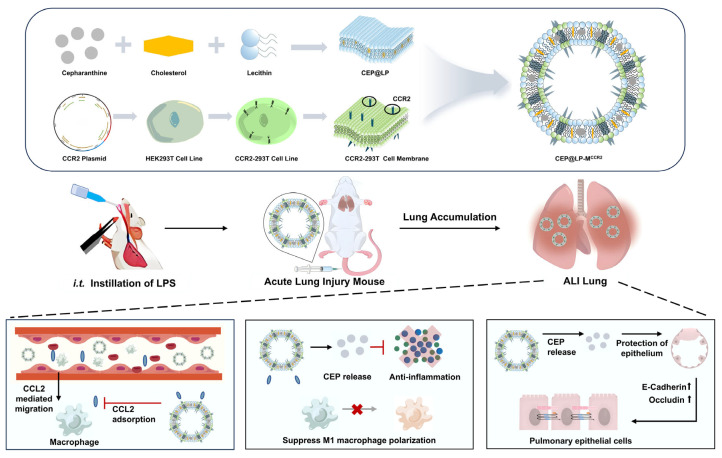
Development of CEP@LP-M^CCR2^ for potential targeted attenuation of ALI.

Based on the above, in this study, CCR2-expressing cell membrane-camouflaged CEP liposomes (CEP@LP-M^CCR2^) as targeted liposomes for potential ALI attenuation were developed (Scheme 1). CEP@LP-M^CCR2^ integrates the biological camouflage of engineered cell membranes with the potential targeting capability of surface-displayed CCR2, which enables accumulation at inflamed lung tissues via the CCL2-CCR2 chemokine axis. CEP@LP-M^CCR2^ exhibits stability, biodistribution, and biocompatibility both in vitro and in vivo. Functionally, the liposomes can ameliorate features of ALI, including pulmonary edema, pro-inflammatory states, and suppression of M1 polarization. Furthermore, RNA sequencing analysis implied CEP@LP-M^CCR2^ may alleviate ALI through the TNF/NF-κB pathway. In all, this work not only provides a potential targeting approach but also introduces an engineered cell membrane strategy that overcomes the limitations of natural membrane-based liposomes, thereby offering a novel approach for ALI attenuation.

## 2. Materials and Methods

### 2.1. Cell Culture and Animals

HEK293T, RAW264.7, BEAS-2B, and MLE-12 were obtained from the National Collection of Authenticated Cell Cultures. Cells were maintained in a humidified incubator at 37 °C with 5% CO_2_. Culture media consisted of DMEM and 1640 medium supplemented with 10% FBS and 1% penicillin–streptomycin.

Male 6–8-week-old BAL/bc mice were purchased from SPF Biotechnology Co., Ltd. (Beijing, China) and maintained in a sterile environment at 24 °C under a 12 h light/dark cycle. All experiments involving animals were approved by the Animal Care and Use Committee of Chengdu University of Traditional Chinese Medicine (Permit No. 2025290).

### 2.2. Construction of CCR2 Overexpressing Cell Line

The CCR2 gene was cloned into the pCDH-CMV-CCR2-GFP-Puro plasmid. HEK293T cells were transfected with Lipofectamine™ 3000 (Thermo Fisher Scientific, Carlsbad, CA, USA), cultured for 48 h and selected with puromycin to establish a stable CCR2-GFP-expressing cell line. The CCR2-293T overexpression cell line was validated by qPCR, Western blot, flow cytometry, and laser confocal microscopy. Plasmid information is provided in Table S1.

### 2.3. Preparation of CEP@LP and CEP@LP-M^CCR2^

To extract of cell membranes, we prepared TM buffer. CCR2-293T cell pellets, were collected, washed twice with PBS, and resuspended in buffer. The sample was sonicated with a probe sonicator for 5 min (100 W, 2 s on, 3 s off), and the supernatant was centrifuged at 2000× *g* for 10 min (to remove nuclei and organelles) and then at 14,000 × *g* for 30 min. The pellet was collected as pretreated CCR2-293T cell membranes (CCR2-293T CM) and lyophilized for storage at −80 °C for future use. Total protein concentrations in CCR2-293T cells and cell membranes were quantified using NanoDrop (Thermo Scientific, Waltham, MA, USA), analyzed via SDS-PAGE and Western blotting.

CEP@LPs were initially prepared via the thin-film dispersion method. The obtained CEP@LPs were mixed with CCR2-293T CM and treated with 100 W ultrasonication for 20 min. After centrifuging the mixture at 12,000× *g* for 15 min and washing twice with distilled water, CEP@LP-M^CCR2^ was obtained.

### 2.4. Characterization of CEP@LP, CEP@LP-M^CCR2^

For encapsulation efficiency and drug loading: 1 mL CEP@LP was processed through dilution in acetonitrile, sonication, and 0.45 μm membrane filtration. Additionally, 1 mL of CEP@LP was placed into an ultrafiltration centrifuge tube, centrifuged at 10,000 rpm for 10 min. The filtrate was collected for UPLC analysis (Agilent 1290 Infinity III, Santa Clara, CA, USA). The liquid in the upper pipe was cepharanthine in liposome (W_Effective_). The liquid in the lower pipe was free drug (W_free_). We calculated EE% and DL%:

EE% = W_Effective_/(W_Effective_ + W_free_); DL% = EE% × W_Effective_/(W_Effective_ + W_free_).

For TEM observation, the sample preparation was performed as follows: A piece of sealing film was laid flat on the surface of a Petri dish. The mesh slide was then placed on the sealed film to ensure stable positioning. A 10 μL aliquot of the sample suspension was added onto the mesh slide using a micropipette. The suspension was allowed to adsorb onto the mesh for 10 min at room temperature, after which the excess liquid was gently blotted dry with filter paper to avoid damaging the sample layer. Subsequently, a 10 μL drop of the staining solution was applied to the mesh slide via a micropipette. The sample was incubated with the staining solution for 2 min to achieve sufficient staining, followed by blotting away the residual staining solution using filter paper. The mesh slide was air-dried in a dust-free environment for 10–20 min until complete desiccation was attained. Finally, the dried mesh slide was mounted onto the TEM sample holder, and morphological observation was conducted using an FEI Tecnai G2 Spirit transmission electron microscope (FEI, Hillsboro, OR, USA).

Dynamic light scattering (DLS), alternatively termed Photon Correlation Spectroscopy (PCS) [29], is a simple technique for investigating the size of nanoscale particles. The detection principle of DLS is based on Brownian motion of suspended particles, which arises from collisions with liquid molecules and induces fluctuations in scattered light intensity. By analyzing such light scattering signals, DLS enables the determination of the average particle size of the target sample [29]. Dynamic light scattering was employed to measure the diameter, PDI, and zeta (BeNano 90 Zeta, Dandong, China). For the physical stability assessment of liposomes, the liposomes solution was equally divided into seven vials, labeled as Day0 to Day7. All sample vials were placed in ice at 4 °C in the dark. The Day0 group was tested immediately, while the remaining groups were stored for the corresponding periods. Particle size, PDI and zeta potential were measured at the respective time points.

### 2.5. Biosafety Assessment of CEP@LP-M^CCR2^

For hemolysis rate (HR): From fresh rat blood, a 2% red blood cell (RBC) suspension was prepared. The RBCs were incubated with various concentrations of LP and LP-M at 37 °C for 1 h, using H_2_O and PBS as positive and negative controls, respectively. After centrifugation, the optical density (OD) of the supernatant was measured at 540 nm. HR was calculated using the following formula:

HR = (OD _Sample_ − OD _Negative_)/(OD _Positive_ − OD _Negative_) × 100%.

Live/dead cell staining was performed according to the established protocol of the Calcein/Propidium IoDide Cytotoxicity Assay Kit (Beyotime, Shanghai, China). BEAS-2B cells were seeded in 12-well plates. Live cells were stained with Calcein AM, and dead cells were stained with PI. After incubation at 37 °C for 30 min, the cells were washed with PBS again and imaged under an inverted fluorescence microscope at 100× magnification (Olympus CKX53, Shinjuku City, Japan).

The cytotoxicity of the drugs was assessed using CCK-8 assays (TargetMol, Wellesley Hills, MA, USA). RAW264.7 and BEAS-2B cells were seeded at densities of 1 × 10^4^ and 5 × 10^3^ cells/well in 96-well plates, added with different liposomes, and then analyzed. To examine the effects of LPS and CEP on cell growth, the cell suspension was inoculated in a 96-well plate at a density of 5 × 10^3^ BEAS-2B cells per well for 12 h. We added 30 μg/mL LPS to 1640 medium supplemented with 1% FBS and incubated it for 24 h. The subsequent steps are as described above. Detecting CEP reversal of LPS damage is the same as described above.

### 2.6. LPS-Induced Cellular Inflammation

RAW264.7 cells were inoculated in 6-well plates. When growing to 60–70% of confluence, cells were sequentially treated with different drugs for 10–12 h, challenged by 1 μg/mL LPS for 4 h, and incubated again with the drugs for 6 h. Cells were inoculated in 6-well plates.

BEAS-2B, MLE-12 cells were inoculated in 6-well plates. When growing to 60–70% of confluence, cells were sequentially treated with 30 μg/mL and 1 μg/mL LPS for 24 h and 12 h, treated with different drugs for 24 h.

### 2.7. Scratch Assay

Two perpendicular lines were pre-drawn on the back of each well in a 6-well plate using a marker pen. BEAS-2B cells were seeded in logarithmic growth phase at 5 × 10^5^ cells per well into the 6-well plate. Under a microscope, cell confluence was observed until reaching 80%. A tip was used to create scratch marks. PBS was used to wash three times to remove detached cells. We added pre-prepared cell culture medium containing 1% FBS. Different drugs were added for 4 h pretreatment in the incubator. Subsequently, we added LPS and CEP, observed after 24 h. The scratch healing area was quantified.

### 2.8. In Vitro Uptake

To investigate the effect of different membrane protein-to-lecithin mass ratios in CEP@LP-M^CCR2^ on cellular uptake, we employed Did@LP-M^CCR2^ with different membrane protein-to-lecithin mass ratios. MLE-12 cells were seeded at 1 × 10^5^ cells per well in 12-well plates and cultured overnight. Cells were then cultured for 24 h in 1640 medium supplemented with 1% FBS and 30 μg/mL LPS. Different drugs were added to respective cell populations. After 4 h, we collected the cells and prepared a cell suspension. Cell uptake was quantified using a flow cytometer (Agilent Technologies, Santa Clara, CA, USA).

To assess the targeting efficacy of Did@LP-M^CCR2^ in an in vitro inflammatory environment, we similarly employed flow cytometry to quantify uptake. We prepared Did@LP-M^CCR2^. Cells were plated and treated with LPS as described above. Free Did, Did@LP, Did@LP-M, and Did@LP-M^CCR2^ were added to their respective cell populations. The cell uptake method is the same as above.

### 2.9. ALI Animal Model and Pharmacodynamic Studies

At 12 h after LPS intratracheal injection, mice received a tail vein injection (200 μL) of 0.75 mg/mL CEP, CEP@LP, CEP@LP-M^CCR2^, or CEP@LP-M. The control and model groups received an equal volume of PBS. Throughout the experiment, mouse body weight was recorded daily. All mice were euthanized at 36 h post-drug. Bronchoalveolar lavage fluid (BALF), lungs, liver, spleen, heart, kidneys, and blood were collected for further analysis.

A total of 1 mL of BALF was collected, centrifuged for total protein concentration. The cell pellet was subjected to lysing with RBC lysis solution on ice and centrifuged. The newly produced cell pellet was resuspended for cell counting with a cell counter (Countstar, Shanghai, China).

The complete fresh lung tissues from each mouse were harvested and weighed (W_1_). The relative murine body weight was recorded at the time of sacrifice (W_2_). The lung index was determined by the following formula:

Lung index = W_1_/W_2_ × 100%.

The histopathology of murine tissues was evaluated by H&E staining. Fresh specimens of lungs, hearts, livers, kidneys, and spleens were immersed in a 4% paraformaldehyde solution for a minimum of 24 h, subsequently embedded in paraffin. The resulting sections were affixed to slides, desiccated, and subjected to H&E staining following established procedures. In the case of the lung tissues, the images were taken by use of an automatic 138 digital slide scanner (Pannoramic MIDI, 3DHISTECH, Budapest, Hungary) and analyzed using CaseViewer software 2.4.

### 2.10. In Vivo Imaging System (IVIS)

To observe the in vivo distribution and lung targeting properties, we first established an ALI animal model in male BAL/bc mice by LPS. After 12 h, a tail vein injection of Did, Did@LP, Did@LP-M, and Did@LP-M^CCR2^ (equivalent in volume to the aforementioned CEP@LP) was administered. After imaging at 2, 6, 12, and 24 h, mice were euthanized; lung, liver, heart, spleen, and kidneys were collected. Imaging was performed using Multifunctional imaging system (iBox Scientia, Analytik Jena, Jena, Germany). Data were analyzed based on fluorescence intensity.

### 2.11. RNA Sequencing and Data Analysis

Total RNA was extracted from lung tissue and reverse transcribed into cDNA; PCR products were denatured into single strands and then sequenced on the DNBSEQ-T7 platform (BGI, Shenzhen, China). Raw FASTQ data were filtered and aligned to the reference genome. Differential gene expression analysis was then performed using DESeq2. The resulting differentially expressed genes (DEGs) were subjected to functional enrichment analysis using the GO and KEGG databases. Visualizations were generated to interpret the results. A heatmap was plotted by https://www.bioinformatics.com.cn (accessed on 4 November 2025), an online platform for data analysis and visualization.

### 2.12. Molecular Docking

The structures of the target proteins (TNFR1, IκBα, NF-κB p65) were acquired from the PDB and prepared with PyMOL 2.4.0. The structure of CEP was obtained from PubChem and prepared with AutoDock Tools 1.1.2. Molecular docking was performed between the proteins and CEP with AutoDock Vina 1.5.6.

### 2.13. Quantitative Real-Time PCR

Following the manufacturer’s instructions, total RNA was extracted from cells using TRIzol reagent (Takara, Kyoto, Japan). RNA concentration was measured using a NanoDrop, and RNA quality was assessed by agarose gel electrophoresis. Subsequently, complementary DNA (cDNA) was synthesized using the PrimeScript™ FAST RT Kit with gDNA Eraser. qRT-PCR was performed using TB Green Premix ExTaqTM II on a QuantStudio 5 Real-Time PCR System (Thermo Fisher Scientific, USA), with three biological replicates per PCR reaction. Relative gene expression levels in different cell types were calculated using the 2^−ΔΔCT^ method, with GAPDH as the internal control. Primer sequences were provided by Sangon (Shanghai, China). Primer information is provided in Table S2.

### 2.14. Western Blot

To evaluate the protective effects of CEP@LP-M^CCR2^ on endothelial and epithelial barrier integrity in vitro, BEAS-2B cells were cultured in 6-well plates until reaching 90% confluence. Then, cells were stimulated LPS and different drugs for 24 h. NanoDrop was used to determine total extracted protein concentration. Obtained protein samples were boiled with loading buffer at 95 °C for 10 min, followed by separation via SDS-PAGE. Subsequently, the separated proteins were transferred onto membranes, which were then blocked with 5% non-fat milk for 1 h and incubated overnight at 4 °C with E-cadherin (1:60,000, 20874-1-AP, Proteintech, Wuhan, China) and Occludin antibody (1:27,500, 27260-1-AP, Proteintech, Wuhan, China).

Next day, the membranes were incubated with relative HRP-conjugated secondary antibody for 1 h at room temperature. Protein bands on the membranes were finally visualized using a chemiluminescence imaging system (Tanon 5200 Multi, Shanghai, China) using stripping buffer to strip the target protein, re-incubated with GAPDH (1:50,000, 60004-1-lg, Proteintech, Wuhan, China) primary and secondary antibodies, then visualized (Tanon 5200, Shanghai, China).

To confirm CEP@LP-M^CCR2^’s effects on macrophage polarization, RAW264.7 cells were seeded in 6-well culture plates. Growing to 60–70% confluence, the cells were sequentially incubated with different drugs for 10–12 h, challenged by 1 μg/mL LPS for 4 h, and treated again with the drugs for 6 h. After that, total proteins were extracted and subjected to SDS-PAGE and Western blot by the aforementioned method. Primary antibodies used were iNOS (1:5000, YM8628, Immunoway, San Jose, CA, USA) and Arg1 (1:5000, YM8217, Immunoway, USA). The subsequent steps are the same as previously described.

As for in vivo, total proteins were obtained from lung homogenate and tested by Western blot for assessment of lung permeability, macrophage polarization, and the activation of TNF/NF-κB signaling pathway using the aforementioned way. The primary antibodies utilized were E-cadherin, Occludin, iNOS, Arg1, TNF-α (1:2500, 60291-1-Ig, Proteintech, Wuhan, China), Phospho-IκBα (1:5000, YM8850, Immunoway, USA), IκBα (1:5000, YM8511, Immunoway, USA), Phospho-NF-κB p65 (1:1000, YP0191, Immunoway, USA), and NF-κB p65 (1:1000, YM3111, Immunoway, USA). The subsequent steps are the same as previously described.

For specific detection of CCR2, total protein concentrations were quantified with NanoDrop and analyzed by SDS-PAGE and Western blotting. The primary antibodies utilized were CCR2 (1:3000, A2385, ABclonal, Wuhan, China). Internal reference antibody is Sodium Potassium ATPase Antibody (1:7500, ABmart, Shanghai, China).

### 2.15. Statistical Analysis

All experimental data in this study were analyzed and graphs were produced using GraphPad Prism 10.1.2. Data are presented as mean ± standard deviation (SD). Statistical analyses were performed through one-way ANOVA or two-sample *t*-test. Differences between every comparison were considered significant if *p*-value < 0.05.

## 3. Results

### 3.1. Establishment of CCR2-Overexpressing HEK293T Cell Line

HEK293T cells (293T) are a transformed cell line derived from human embryonic kidney cells. They possess a rapid proliferation ability and are easy to cultivate [30], maintain high genetic stability and reduce experimental variations. Therefore, in this study, HEK 293T cells were selected for the preparation of a CCR2-overexpressing HEK293T cell line (CCR2-293T).

**Figure 1:**
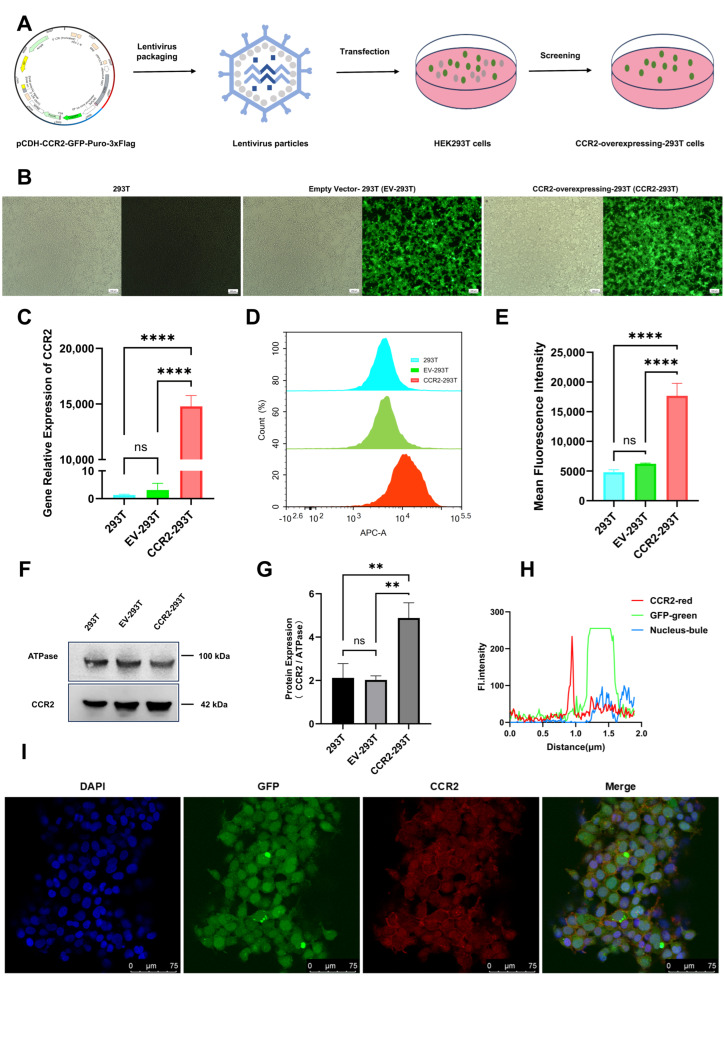
Construction and characterization of CCR2-293T. (**A**) Schematic illustration of the establishment of the CCR2-overexpressing cell line. (**B**) Expression of green fluorescent protein (GFP) as observed by fluorescence microscopy. (**C**) CCR2 mRNA levels in 293T, EV-293T, CCR2- 293T by qRT- PCR. (*n* = 3) (**D**,**E**) Analysis of CCR2 protein by flow cytometry analysis. (*n* = 3) (**F**,**G**) Western blot analysis. (*n* = 3) (**H**) Plot profile for co-localization analysis of CCR2 protein and membrane. (**I**) Distribution of CCR2 protein (green) and membrane (red) in 293T cells. Images were captured using 40× oil immersion lens of confocal microscope. Scale bar, 75 μm. ns, not significant (*p* ≥ 0.05), ** *p* < 0.01, **** *p* < 0.0001.

**Figure A1:**
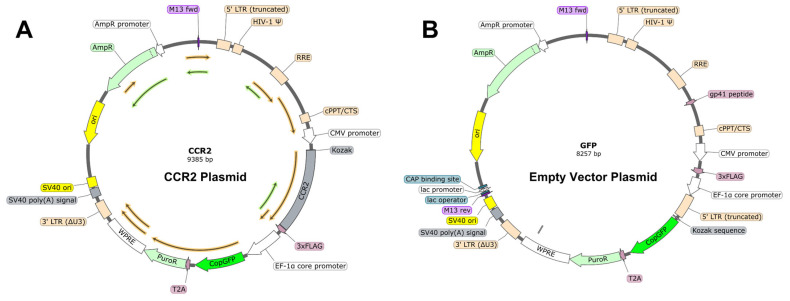
CCR2 plasmid (**A**) and empty vector plasmid (**B**) diagram.

First, the plasmid carrying the CCR2 and GFP gene was used to transfect 293T cells with lentivirus, and then puromycin selection was performed to construct a stable cell line with CCR2 overexpression (CCR2-293T) (Figure 1A and Figure A1). As shown by the localization of the CCR2-GFP construct of green fluorescent protein, CCR2 is expressed and localized on the plasma membrane (Figure 1B). This indicates that the lentiviral vector marked with the reporter gene GFP has successfully been transduced into HEK293T cells, and it also confirms that the CCR2 gene has been effectively transferred into the cells. The untransfected 293T cells do not show green fluorescence.

The qPCR result analysis showed that (Figure 1C), compared with the untransfected cells, the mRNA level of CCR2 in the transfected 293T cells increased, confirming the success of gene integration and transcription. The results of flow cytometry showed that (Figure 1D,E), compared with the untransfected 293T cells and the EV-293T cells that were only transfected with the GFP reporter gene, the average fluorescence intensity of CCR2-293T cells was shifted, indicating that the CCR2 gene was highly expressed in the transfected CCR2-293T cell line. To further validate these findings, we conducted a Western blot analysis of CCR2 protein expression. The results showed that the expression level of CCR2 protein in the transfected cells increased (Figure 1F,G). In addition, we also found that the expression levels of the CCR2 gene and the protein expression levels in the EV-293T cells that were only transfected with the reporter gene showed no significant difference compared to those in the untransfected 293T cells. Finally, we used confocal laser scanning microscopy (CLSM) to observe the co-localized fluorescence signals from the GFP reporter protein (green) and the cell membrane (red) to verify the expression of CCR2 (Figure 1H). We have observed that the fluorescence signal of CCR2-GFP can be detected both on the cell membrane and within the cytoplasm (Figure 1I). This might be attributed to the transport of CCR2-GFP from the cytoplasm to the cell membrane during protein synthesis [31].

These results indicate that the CCR2 gene was integrated, transcribed and translated in the HEK293T cell line; the CCR2 overexpression cell line was successful.

### 3.2. Design and Characterization of CEP@LP-M^CCR2^

**Figure 2:**
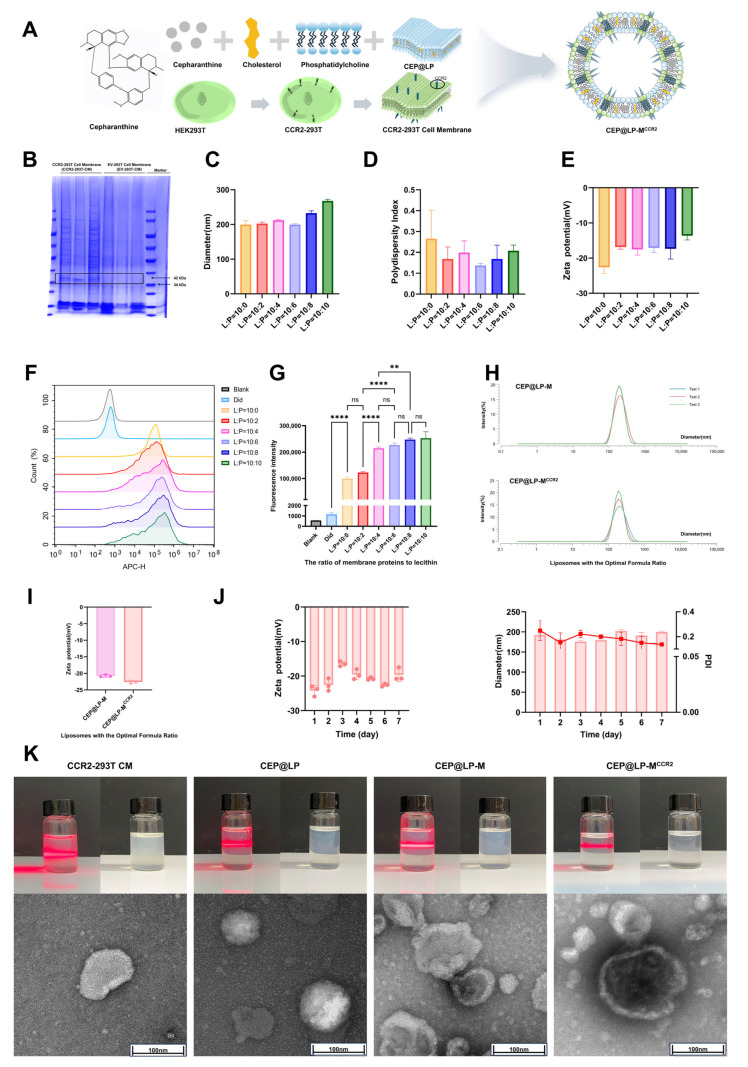
Construction and characterization of CEP@LP-M^CCR2^. (**A**) Schematic illustration of CEP@LP-M^CCR2^ synthesis. (**B**) SDS-PAGE analysis of total membrane proteins collected from CCR2-293T CM, EV-293T CM. (**C**–**G**) Optimization of the ratio of lecithin to membrane proteins. Diameter (**C**), PDI (**D**), zeta (**E**), fluorescence uptake of qualitative analysis (**F**), quantitative analysis (**G**). (**H**,**I**) Representative size distribution and zeta of the formulation. (**J**) Stability of CEP@LP-M^CCR2^ after storage 4 °C. (**K**) Appearance and TEM images. ns, not significant (*p* ≥ 0.05), ** *p* < 0.01, **** *p* < 0.0001.

**Figure A2:**
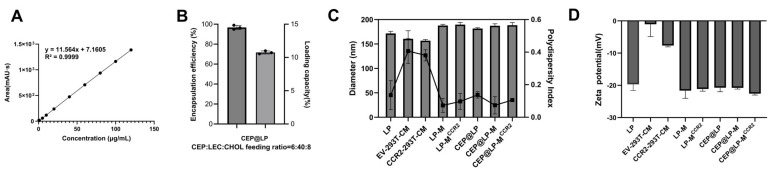
Construction and characterization of CEP@LP. (**A**) Standard curve of CEP. (**B**) Encapsulation efficiency and loading capacity of CEP in LP at a fixed ratio. (**C**,**D**) Diameter, PDI and Zeta for each distinct LP. *n* = 3.

Based on different addition ratios of CEP, lecithin (LEC) and cholesterol (CHOL), we found when the proportions of each component were 6:40:8, the encapsulation rate was the best at 96.7% and the drug loading was 10.7% (Figure A2A,B and Figure S3). We prepared the CEP liposomes (CEP@LP), extracted the cell membrane (CCR2-293T CM) of the CCR2- 293T cell line (Figure 2A), analyzed total membrane proteins by SDS-PAGE (Figure 2B), used the ultrasonic method to make cell membrane coated CEP@LP, and developed a liposome named CEP@LP-M^CCR2^ (Figure 2A).

In order to optimize the drug formulation, we selected different quality ratios of lecithin and membrane proteins to prepare CEP@LP-M^CCR2^. The optimal drug formulation was determined based on multiple indicators such as zeta, diameter, polydispersity (PDI), and uptake efficiency (Figure 2C–G). Due to the 10:4 ratio, CEP@LP-M^CCR2^ was outstanding in many respects. The particle size is suitable for intravenous injection, the cell uptake efficiency is good, and it conserves cell membrane materials. Therefore, the 10:4 ratio was determined to be the optimal one. Finally, CEP@LP-M and CEP@LP-M^CCR2^ measured to be less than 200 nm and more than 20 mV (Figure 2H,I). To assess the stability of CEP@LP-M^CCR2^, their characterizations were monitored a period of 7 days, and their diameter consistently fluctuated about 200 nm, their PDI ranged from 0.1 to 0.3. These results concluded that CEP@LP-M^CCR2^ formulations remained stable for at least 7 days (Figure 2J).

Subsequently, we conducted a comprehensive study on the morphology and size distribution of CEP@LP-M^CCR2^ using transmission electron microscopy (TEM) imaging. Compared to CEP@LP, the outer layer of CEP@LP-M and CEP@LP-M^CCR2^ is covered by a cell membrane. The three entities present a spherical shape. The outer layer of CEP@LP-M and CEP@LP-M^CCR2^ is covered by a layer of cell membrane. The cell membrane vesicles have a typical phospholipid bilayer structure. TEM imaging shows that CEP@LP-M and CEP@LP-M^CCR2^ have unique core–shell structures, thereby confirming the success of the membrane coating [32] (Figure 2K). These data suggest that CEP@LP-M^CCR2^ is suitable for subsequent in vitro and in vivo studies.

### 3.3. In Vitro and In Vivo Targeting of CEP@LP-M^CCR2^

**Figure 3:**
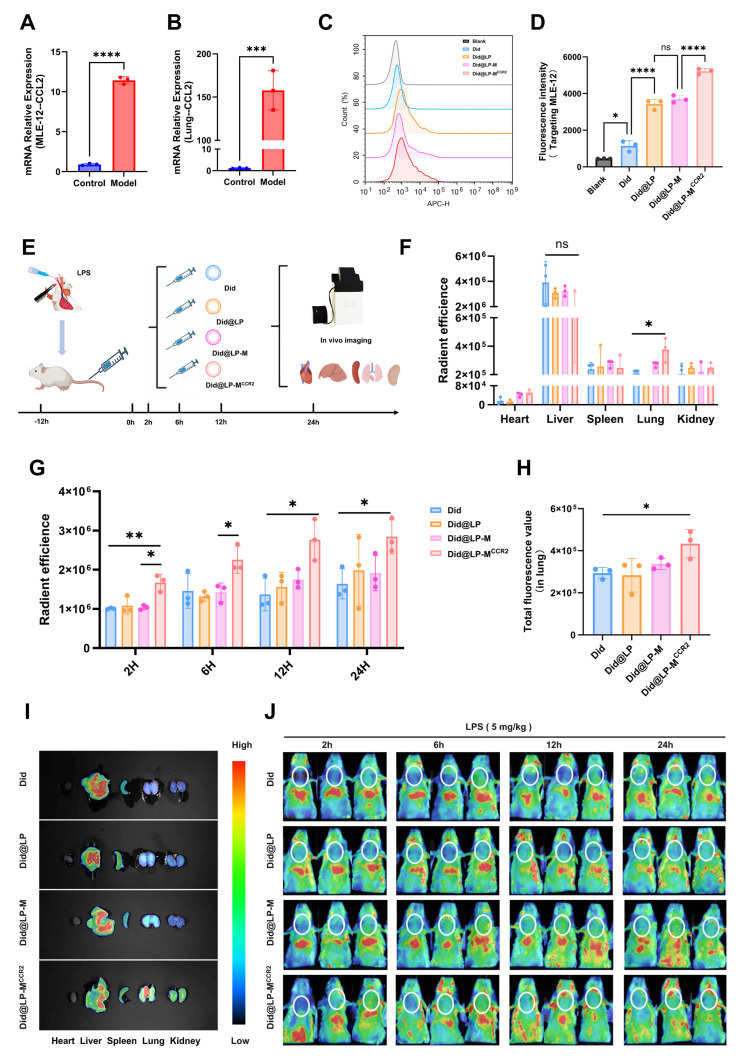
In vivo and in vitro targeting efficacy of CEP@LP-M^CCR2^. (**A**,**B**) Levels of CCL2 in MLE-12 cells and in mouse lung induced by LPS. (**C**,**D**) Targeted uptake in vitro by using flow cytometry. Targeted to MLE-12. (**E**) Schematic illustration of the in vivo imaging schedule for ALI mice. (**F**) Quantitative analysis the fluorescence imaging of ex vivo bioluminescence images of heart, liver, spleen, lung, and kidney tissues obtained 24 h post-injection from parallel images (**I**). (**G**) Quantitative analysis of the fluorescence imaging of mice from parallel images (**J**) at the indicated time points. (**H**) Quantitative analysis of the fluorescence of isolated lung in vitro imaging from parallel images (Figure A3D). (**I**) Imaging of major isolated organs 24 h after tail vein injection. (**J**) Representative whole-body imaging of LPS-induced pneumonia mice at different time points after tail vein injection. ns, not significant (*p* ≥ 0.05), * *p* < 0.05, ** *p* < 0.01, *** *p* < 0.001, **** *p* < 0.0001. The white circle indicates the region for fluorescence quantification in live imaging.

**Figure A3:**
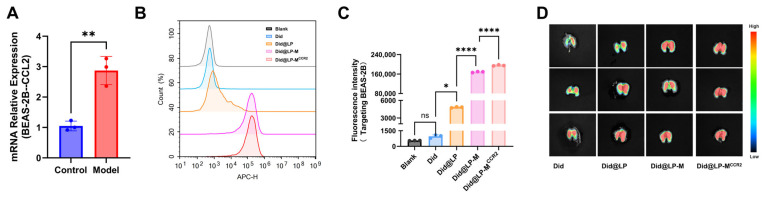
Evaluation of in vitro BEAS-2B cell targeting and in vivo targeting. (**A**) The levels of CCL2 in BEAS-2B cells induced by LPS. (**B**,**C**) Targeted uptake in vitro by using flow cytometry. Targeted to BEAS-2B. (**D**) In vitro imaging of isolated lung tissue. ns, not significant (*p* ≥ 0.05), * *p* < 0.05, ** *p* < 0.01, **** *p* < 0.0001.

The cellular inflammation induced by LPS is a widely recognized method for studying the mechanism of inflammatory responses [33]. The production of CCL2 is one of typical features of the inflammatory microenvironment in ALI. In order to explore whether it can be utilized for specific drug delivery based on the interaction of ligands, we compared the CCL2 levels in normal BEAS-2B, MLE-12, lung tissues and those induced by LPS. Compared with the control group, CCL2 expression in both cells and tissues was increased in the model group 24 h after LPS stimulation (Figure 3A,B and Figure A3A). DiD’ solid (Did) is a lipophilic carbocyanine that can stain the cell membrane and emit red fluorescence. Did can be used for in vivo imaging and the study of fluorescence intensity during in vitro uptake. In NLP, we inserted Did in the same way as in CEP, and separately prepared Did, Did@LP, Did@LP-M, and Did@LP-M^CCR2^.

In vitro, we conducted a quantitative study on the in vitro targeted uptake of Did, Did@LP, Did@LP-M, and Did@LP-M^CCR2^ using flow cytometry. Compared to Did, Did@LP-M^CCR2^ exhibits a 4.56-fold increase in cellular uptake in MLE-12 (Figure 3C,D). Whether it is BEAS-2B or MLE-12, the average fluorescence intensity of Did@LP-M^CCR2^ is different from that of Did@LP (Figure 3C,D and Figure A3B,C). This indicates that nanoparticles encapsulated by the cell membrane are more easily taken up by the cells. The average fluorescence intensity of Did@LP-M^CCR2^ was different from that of Did or Did@LP or Did@LP-M. In an inflammatory environment, when CCL2 is highly expressed in the damaged tissue, the CCR2 protein in Did@LP-M^CCR2^ will promote the accumulation of nanoparticles at the damaged tissue site.

In vivo, the efficiency of drug delivery to lung tissue is crucial for the treatment of ALI. To further evaluate the lung-targeting properties of Did@LP-M^CCR2^ in ALI mice, we injected the nanomedicine (based on an equal dose of Did) into the ALI mouse model stimulated by LPS (5 mg/kg) via the tail vein (Figure 3E).

At 2 h, 6 h, 12 h, and 24 h after tail vein injection, the distribution of Did, Did@LP, Did@LP-M and Did@LP-M^CCR2^ in major organs was observed by in vivo imaging system (Figure 3J). The results indicated that from 2 h to 24 h, the distribution of Did@LP in various organs began to exceed that of free Did. Fluorescence was mainly present in the liver and lungs, and there were also many distributions in the spleen and kidneys, with the least distribution in the heart (Figure 3F,G,I). This might be due to liver metabolism and lung targeting. Over time, Did@LP-M^CCR2^ accumulates more and more in the lungs. Through semi-quantitative analysis of pulmonary fluorescence, the fluorescence intensity accumulated by nanoparticles in the lungs gradually increased with the increase of time, and the fluorescence intensity of each group reached its strongest at 24 h (Figure 3G). Compared to Did, Did@LP-M^CCR2^ exhibits a 1.73-fold increase in lung accumulation in 24 h in vivo (Figure 3H). Furthermore, compared with Did, Did@LP, and Did@LP-M, the fluorescence intensity of Did@LP-M^CCR2^ distributed in the lungs is stronger (Figure 3G,H and Figure A3D). All of these indicate that in LPS-induced ALI mice, due to the increased secretion of chemokine CCL2, Did@LP-M^CCR2^ has better targeting of the lungs. This is of great significance for the treatment of lung diseases, as it can increase the concentration of lung drugs and enhance the therapeutic effect. This might be based on the mutual recognition of ligands between the 293T cell membrane and the damaged vascular endothelium, as well as the mutual recognition and binding of chemokine receptor ligands of CCL2 and CCR2, which promotes the targeted aggregation of Did@LP-M^CCR2^ at the inflammatory site of the lungs.

However, the fluorescence signal at the liver shows the opposite trend (Figure 3F,I). Compared with the Did@LP, the fluorescence intensity of the Did@LP-M^CCR2^ and the Did@LP-M in the liver was lower, suggesting that cell membrane biomimetic nanoparticles might reduce liver metabolism and lower the risk of non-target distribution throughout the body. Meanwhile, compared with other organs, the fluorescence intensity in the liver indicates that a large number of nanomedicines are metabolized through the liver.

### 3.4. Biosafety Assessment of CEP@LP-M^CCR2^

CEP@LP-M^CCR2^ has been proven promising for the treatment of ALI. We further evaluated the potential toxic effects induced by CEP@LP-M^CCR2^.

**Figure A4:**
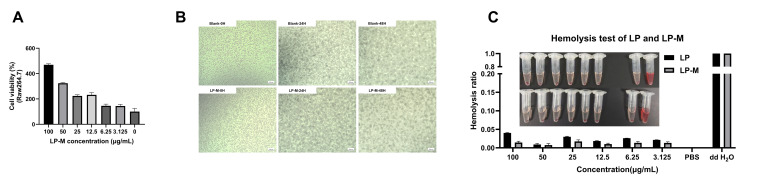
Good biocompatibility of LP-M and LP in vitro. (**A**) CCK-8 assays in RAW264.7. (**B**) Observation of RAW264.7 status. (**C**) Hemolysis ratio assays.

First, we investigated whether the blank nanocarrier (LP-M) without CEP had potential toxicity. CCK-8 assays showed LP-M unaffected RAW264.7 cell viability after 24 or 48 h of treatment (Figure A4A,B). The hemolysis rates of the LP and LP-M groups with the highest dose of 100 μg/mL were calculated to be 4.01% and 1.43% respectively; the nanocarriers were still less than 5% stipulated in the national pharmaceutical industry standard, which indicates that the LP-M and LP groups have good blood compatibility (Figure A4C).

**Figure 4:**
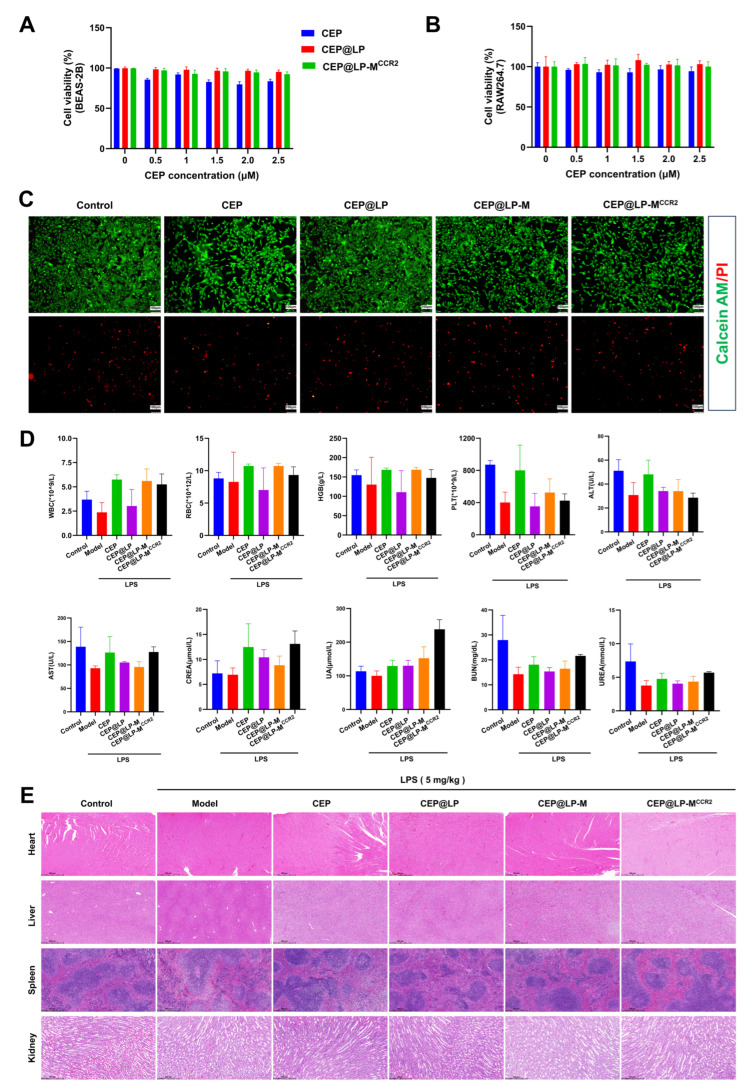
Good biocompatibility of CEP@LP-M^CCR2^ in vitro and in vivo. (**A**,**B**) CCK-8 assays in RAW264.7 and BEAS-2B. (*n* = 3) (**C**) Live/dead cell staining in BEAS-2B. Green: Calcein AM staining for live cells. Red: propidium iodide staining for dead cells. Scale bar, 100 μm. (**D**) Analysis of complete blood count in mice following injection of different drugs. (*n* = 3) (**E**) Histopathological evaluation of heart, liver, spleen, and kidney of LPS-induced ALI mice using H&E staining. Scale bar, 400 μm.

The biocompatibility and drug toxicity of CEP@LP-M^CCR2^ were evaluated using CCK-8 assays and live/dead staining. In both RAW264.7 and BEAS-2B cells, the CEP@LP-M^CCR2^ and CEP@LP groups showed higher cell survival rates than the CEP group. These results indicate that liposome and cell membrane coating reduced the cytotoxicity of CEP (Figure 4A,B). Live/dead staining showed that almost 90% of the live cells (Calcium AM, green) and a small number of dead cells (Propidium Iodide, red) indicated that CEP@LP-M and CEP@LP-M^CCR2^ had good biocompatibility in vitro (Figure 4C).

Evaluate in vivo biocompatibility, we performed a complete blood count and hematoxylin and eosin staining (H&E). Complete blood cell counts showed that there were no significant changes in platelet (PLT), red blood cell (RBC), white blood cell (WBC) counts, or hemoglobin (HGB) levels in mice treated with nanoparticles. This indicates that CEP@LP-M and CEP@LP-MCCR2 do not have obvious adverse effects on the hematopoietic system, nor do they induce abnormal changes in blood components. In addition, we detected biomarkers of liver and kidney function; nanoparticle treatment had no significant effect on the levels of alanine aminotransferase (ALT), aspartate aminotransferase (AST), or creatinine (CR) (Figure 4D). H&E staining of the tissue sections showed that the nanoparticles did not cause significant tissue damage to major organs such as the heart, liver, spleen and kidneys (Figure 4E).

These results indicate that CEP@LP-M and CEP@LP-M^CCR2^ have good biocompatibility and no obvious tissue toxicity; they could provide an important safety foundation for further research.

### 3.5. Protection of CEP@LP-M^CCR2^ on Epithelial Integrity In Vitro

The airway epithelial barrier formed by intercellular junctions (e.g., tight junctions, gap junctions, adhesion junctions) [34,35] plays a role in preventing inhalation of various airborne pollutants and modulates inflammation [36,37]. During ALI, pro-inflammatory cytokines can induce and aggravate airway epithelial cell dysfunction and disrupt their tight junctions. This can lead to pulmonary edema, and even death. Therefore, suppressing inflammation and restoring the function of the airway epithelial barrier may help improve symptoms of ALI [38,39,40,41].

**Figure 5:**
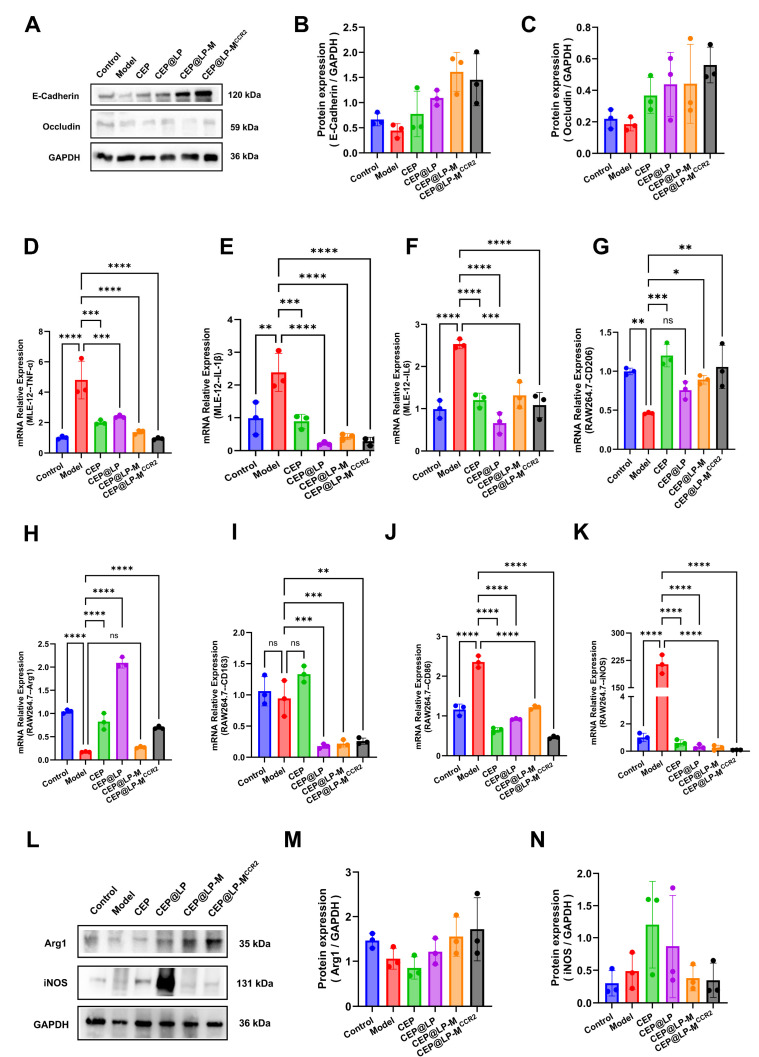
Effects of CEP@LP-M^CCR2^ in vitro. (**A**–**C**) Western blot analysis protective effects of CEP@LP-M^CCR2^ on epithelial integrity in BEAS-2B. (**D**–**F**) The mRNA expression of TNF-α, IL6, IL1β in MLE-12 by qRT-PCR. (**G**–**K**) The mRNA expression of CD206 (M2), Arg1 (M2), CD163 (M2), iNOS (M1), CD86 (M1) in RAW264.7 by qRT-PCR. Internal control: GAPDH. (**L**–**N**) Protein expression of Arg1 and iNOS in RAW264.7 cells by Western blot. Statistical analysis of proteins calculated by ImageJ. Internal control: GAPDH. ns, not significant (*p* ≥ 0.05), * *p* < 0.05, ** *p* < 0.01, *** *p* < 0.001, **** *p* < 0.0001.

**Figure A5:**
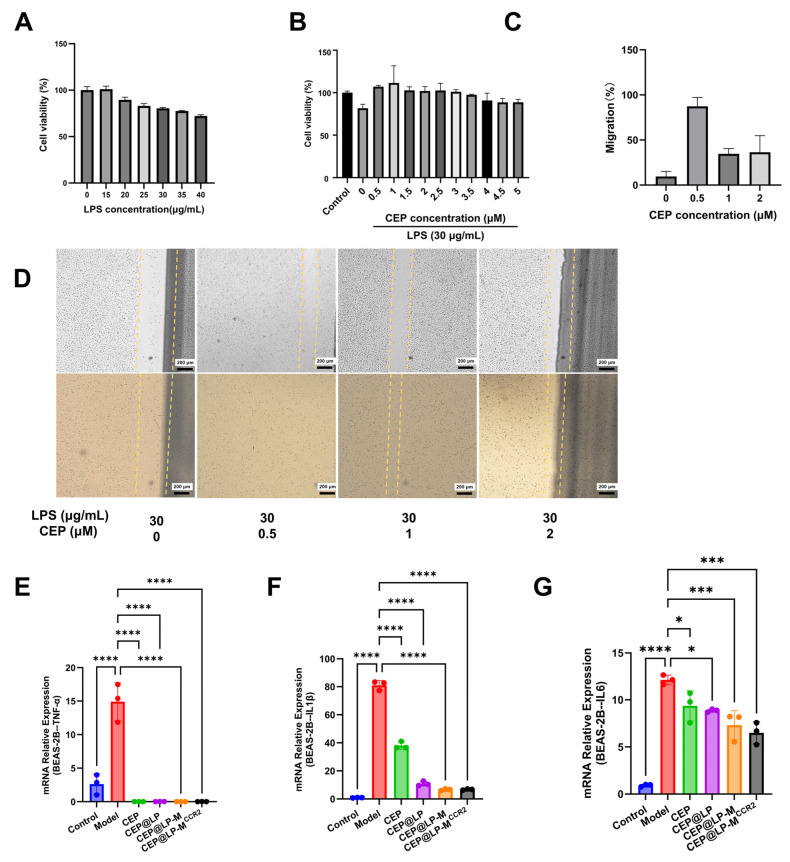
Protection of CEP, CEP@LP-M^CCR2^ reduces inflammation. (**A**) Cell viability analysis of BEAS-2B cells treated with different doses of LPS for 24 h. (**B**) The effect of CEP on the viability of LPS-treated BEAS-2B cells. (**C**,**D**) Effect of CEP on the migration of BEAS-2B cells. *n* = 3. (**E**–**G**) mRNA expression of TNF-α, IL-1β, IL-6 in BEAS-2B by qRT-PCR. Internal control: GAPDH. ns, not significant (*p* ≥ 0.05), * *p* < 0.05, *** *p* < 0.001, **** *p* < 0.0001.

To test whether CEP is effective, we first used LPS-stimulated BEAS-2B; after treatment with 30 μg/mL LPS for 24 h, the cell viability decreased by 19% (Figure A5A). However, CEP can inhibit the damage of LPS to BEAS-2B cells. Compared with the LPS treatment group alone, the viability of BEAS-2B cells could be improved by 0.5 μM CEP (Figure A5B). We employed a scratch assay to determine whether CEP can repair lung epithelial cells damaged by inflammation. Compared with the control group, LPS inhibited cells migration; 0.5 μM CEP enhanced BEAS-2B cells migration. It is suggested that CEP may promote the migration of pulmonary epithelial cells (Figure A5C,D). To assess the potential protective effects of CEP@LP-M^CCR2^ on epithelial integrity, following stimulation of BEAS-2B cells exposed to LPS and CEP, we performed Western blot analysis. Observations revealed that E-cadherin and Occludin expression levels were lower in the model group than in the control group. This reduction trend was reversed after drug administration (Figure 5A–C). These findings suggest that CEP@LP-M^CCR2^ may possess reparative effects in vitro.

### 3.6. CEP@LP-M^CCR2^ Reduces Inflammation and Suppresses M1 Polarization In Vitro

We used qRT-PCR to detect the secretion of inflammatory factors in MLE-12 cells (Figure 5D–F) and BEAS-2B cells (Figure A5E–G) stimulated by LPS respectively. It was found that CEP could reduce the increase of TNF-α, IL6 and ILβ caused by LPS, and the effect of CEP@LP-M^CCR2^ was better than that of CEP. This indicates that CEP@LP-M^CCR2^ could reduce effects on inflammation.

Studies have shown that in ALI, damaged lung epithelial cells secrete large amounts of the chemokine CCL2, which recruits monocyte-derived macrophages expressing the CCR2 receptor. Peripheral circulation monocytes can differentiate into interstitial macrophages and alveolar macrophages [42], while tissue macrophages can further differentiate into alveolar macrophages [42], which in turn induces aberrant activation of macrophages.

In vitro studies have shown that alveolar macrophages derived from monocytes respond to lipopolysaccharide stimulation by secreting a relatively large amount of keratinocyte chemokines and various cytokines, promoting the chemotaxis of alveolar macrophages derived from monocytes to lung tissue and facilitating the occurrence and development of inflammation [43]. We established an in vitro model of macrophage polarization by stimulating RAW246.7 with LPS and detected the cell markers in RAW246.7 by qPCR. In this experiment, we selected markers for M1 macrophages (such as CD86 and iNOS) and markers for M2 macrophages (such as Arg1, CD163, and CD206) [44,45,46]. We found that LPS induces RAW246.7 to express M1-type macrophage markers, while CEP inhibits the expression of CD86 and iNOS (Figure 5J,K) and increases the expression of CD206 and Arg1 (Figure 5G,H). Interestingly, we found that CD163, as a marker of M2-type macrophages, reduced its expression after CEP@LP, CEP@LP-M, and CEP@LP-M^CCR2^ treatment (Figure 5I). We speculate that this may be due to a compensatory mechanism resulting from the downregulation of other genes mediating macrophage activation and polarization. For M2-type polarization, only a minor effect is observed. The protein expressions of M1/M2-associated genes were assessed by Western blot. Results showed that compared to the control, the protein expression of iNOS was upregulated in the model group, and this reduction trend was reversed by the addition of the drugs. The protein expression pattern of Arg1 was opposite to that of iNOS (Figure 5L–N).

### 3.7. The Potential Protective Effects of CEP@LP-M^CCR2^ In Vivo

**Figure 6:**
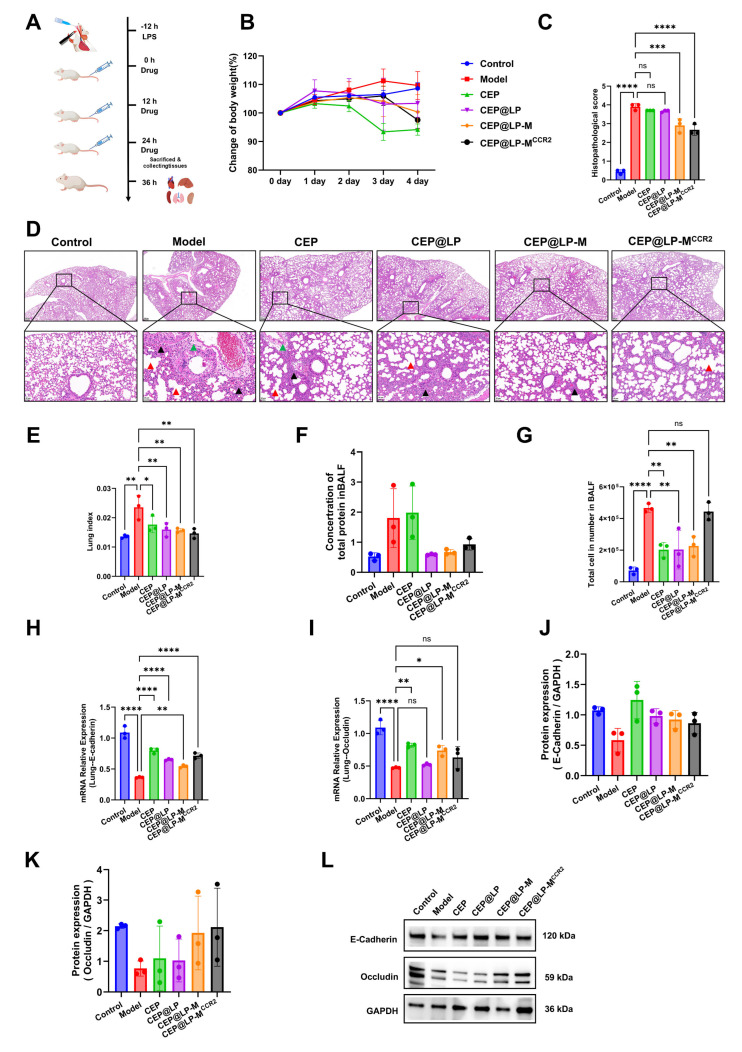
Protective effects of CEP@LP-M^CCR2^ on LPS-induced ALI in vivo. (**A**) Schematic illustrating of animal experimental design. (**B**) Changes of murine body weight loss among all groups for 5 d (*n* = 3). (**C**) Pathological scores of H&E staining results. (**D**) Histopathology of lung tissues by H&E staining. Scale bar, 200 μm, 50 μm. The black arrows, inflammatory cells. The red arrows are the destruction of alveolar structure. The green arrows are the exudate of blood. (**E**) Lung index of mice. (**F**) Protein concentration in BALF. (**G**) Total cell counts in BALF. (**H**,**I**) The normalized mRNA expression of E-Cadherin and Occludin by qRT-PCR. Internal control, GAPDH. (**J**–**L**) Protein expression of E-Cadherin and Occludin protein by Western blot. Normalized protein expressions calculated by using ImageJ software 1.54f. Internal control: GAPDH. ns, not significant (*p* ≥ 0.05), * *p* < 0.05, ** *p* < 0.01, *** *p* < 0.001, **** *p* < 0.0001.

After confirming the role of CEP@LP-M^CCR2^ in vitro, we further verified the therapeutic effect of CEP@LP-M^CCR2^ on ALI models. We established a mouse model of acute lung injury (ALI) by endotoxin infusion into the trachea and evaluated its effects by tail vein injection of CEP, CEP@LP, CEP@LP-M, and CEP@LP-M^CCR2^ (Figure 6A). Body weight loss is a key index of numerous diseases including ALI. The CEP@LP-M^CCR2^ group exhibited lower weight loss compared to the CEP group (Figure 6B). In the histopathological analysis, it was observed that the model group showed obvious inflammatory cell infiltration, alveolar structure destruction and bloody exudation, while the injection of CEP, CEP@LP, CEP@LP-M and CEP@LP-M^CCR2^ could alleviate these changes (Figure 6D). Based on H&E staining, pathological scores showed that CEP@LP-M^CCR2^ could attenuate lung injury in ALI mice (Figure 6C). Moreover, lung index, the total protein concentration and total cell number in the BALF were decreased in the model group and relieved by injection of the drugs, suggesting that CEP@LP-M^CCR2^ mitigates LPS-induced damage to lung (Figure 6E–G). It indicates that after treatment, the leakage of cells and proteins can be decreased, and it has a potential protective effect on lung tissue.

As the main tight junction proteins, E-Cadherin and Occludin play a very important role in maintaining the airway epithelial barrier function [47]. We analyzed the epithelial barrier markers in lung tissue by qRT-PCR (Figure 6H,I) and Western blot (Figure 6J–L). The research results show that LPS reduces the protein levels of E-Cadherin and Occludin in lung tissue and disrupts the barrier function composed of them. However, CEP, CEP@LP, CEP@LP-M, and CEP@LP-M^CCR2^ can all improve barrier dysfunction caused by LPS. It indicates that CEP@LP-M^CCR2^ has a potential protective effect on the barrier function of airway epithelial cells caused by LPS.

In conclusion, these results indicate that CEP@LP-M^CCR2^ has potential therapeutic advantages in the treatment of ALI.

### 3.8. CEP@LP-M^CCR2^’s Effects on Balancing Macrophage Polarization In Vivo

The balance of M1/M2 macrophage polarization plays a crucial role throughout the progression of ALI, including the regulation of inflammatory states, the rehabilitation of lung injury, and the repair of the lung barrier [48]. During the acute phase of ALI, pro-inflammatory M1 macrophages increase and release inflammatory factors such as TNF-α, IL-6 and IL-1β, leading to uncontrolled inflammation, lung injury and barrier destruction. During the recovery period of infection, macrophages transform from monocytes into M2 type and express arginase-1, which can stimulate tissue repairment and cell growth [49].

**Figure 7:**
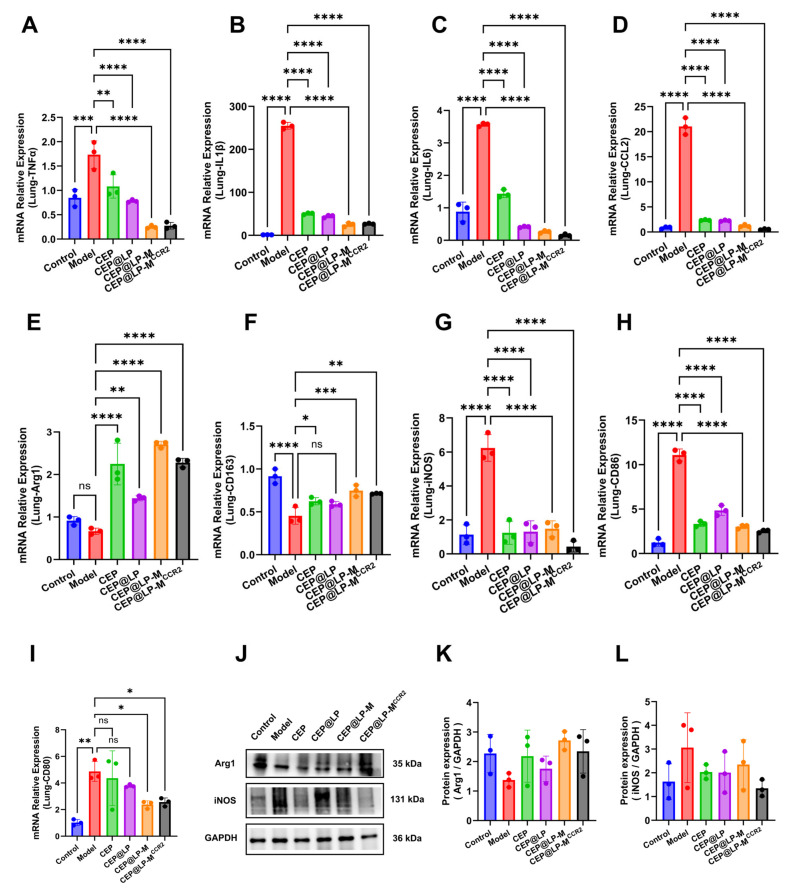
CEP@LP-M^CCR2^ on reducing inflammation and maintaining balance of macrophage polarization in lungs of ALI mice. (**A**–**I**) The normalized transcriptional expressions of M1-associated genes (TNF-α, IL-1β, IL-6, CCL2, iNOS, CD86, CD80) and M2-associated genes (Arg1, CD163) by qRT-PCR. Internal control: GAPDH. (**J**) Western blot of iNOS (M1) and Arg1 (M2). (**K**,**L**) Statistical analysis of proteins calculated by Image J. Internal control: GAPDH. ns, not significant (*p* ≥ 0.05), * *p* < 0.05, ** *p* < 0.01, *** *p* < 0.001, **** *p* < 0.0001.

To verify the effect of CEP@LP-M^CCR2^ on maintaining M1/M2 macrophage polarization, we conducted the expression genes of M1 and M2 phenotypic markers in lung tissue by qRT-PCR. It was found that at the transcriptional level, compared with the model, the transcriptional expression of M1-related TNF-α, IL-6, IL-1β, iNOS, CD86, and CD80 [50] was downregulated in the drug treatment group (Figure 7A–C,G–I), while the mRNA of M2-related Arg1 and CD163 changed in the opposite way (Figure 7E,F). Downregulation of CCL2 mRNA may demonstrate the dual role of CEP@LP-M^CCR2^: first, CCR2-293T CM may bind CCL2 in ALI; second, CEP can downregulate M1-related genes (Figure 7D). Western blot analysis showed that iNOS protein was downregulated and Arg1 protein elevated by inhalation of the drugs (Figure 7J–L).

In conclusion, these findings support the potential therapeutic benefits of CEP@LP-M^CCR2^ in regulating macrophage polarization and alleviating lung inflammation in ALI.

### 3.9. Overall Therapeutic Effects of CEP@LP-M^CCR2^ on ALI by RNA-Seq In Vivo

To further clarify the overall therapeutic effect of CEP@LP-M^CCR2^, the transcriptional profile of lung tissue was established using RNA-seq analysis.

**Figure A6:**
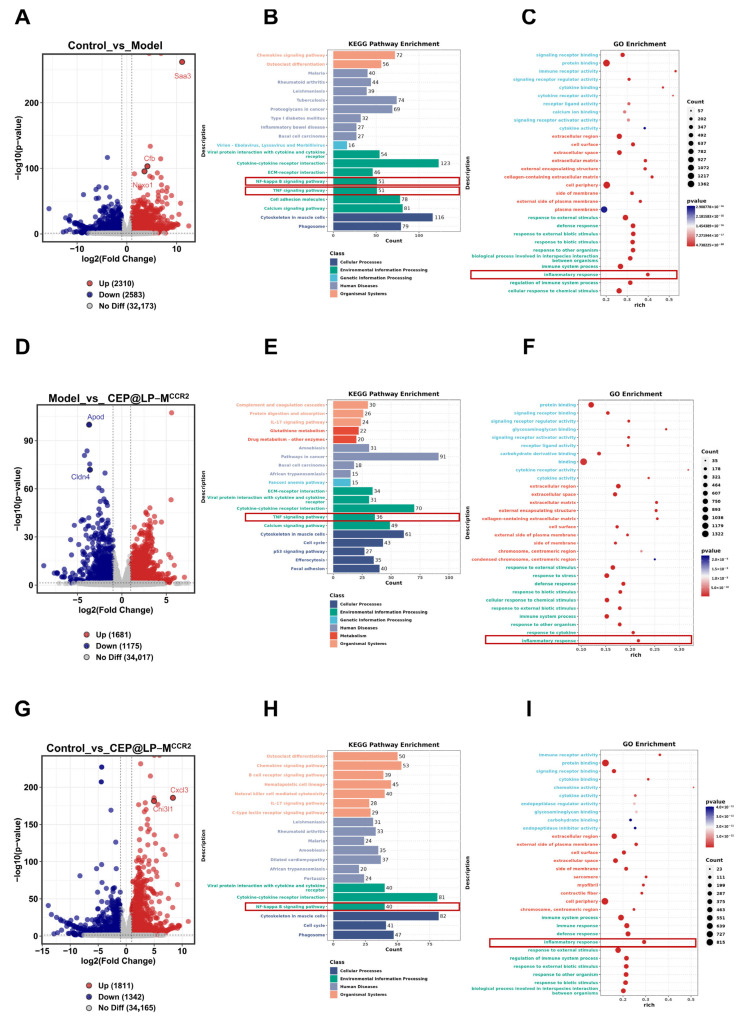
Transcriptome sequencing analysis of LPS-induced lung injury mice treated with CEP@LP-M^CCR2^. (**A**–**C**) Control vs. Model. (**D**–**F**) Model vs. CEP@LP-M^CCR2^. (**G**–**I**) Control vs. CEP@LP-M^CCR2^. Signal pathways of particular interest are highlighted with red boxes.

We compared the model group with the control group and found that 2310 genes were upregulated and 2583 genes were downregulated. *Saa3*, *Cfb*, and *Noxo1* were directly associated with inflammation [51,52,53], indicating that inflammatory responses were occurring at the injury sites of ALI following LPS treatment (Figure A6A). These differentially expressed genes (DEGs).

Comparing the model group with CEP@LP-M^CCR2^ revealed 1681 genes were upregulated and 1175 were downregulated (Figure A6D). The anti-inflammatory protective factors *Apod* was downregulated, along with *Cldn4*, which is associated with tight junctions [54]. This indicates that the inflammatory environment caused by acute LPS-induced lung injury impaired the lung’s barrier function.

We compared the control group with CEP@LP-M^CCR2^ treatment group identified 1811 genes that were upregulated and 1342 genes that were downregulated (Figure A6G), including chemokine *Cxcl3*, inflammatory factor *Chi3l1* and so on.

KEGG analyses indicated that these differentially expressed genes (DEGs) tended to be enriched in pathways including the NF-κB signaling pathway and TNF signaling pathway (Figure A6B,E,H). Meanwhile, GO analyses suggested that the functional annotations across all groups were more prominently concentrated in inflammatory responses (Figure A6C,F,I).

**Figure 8:**
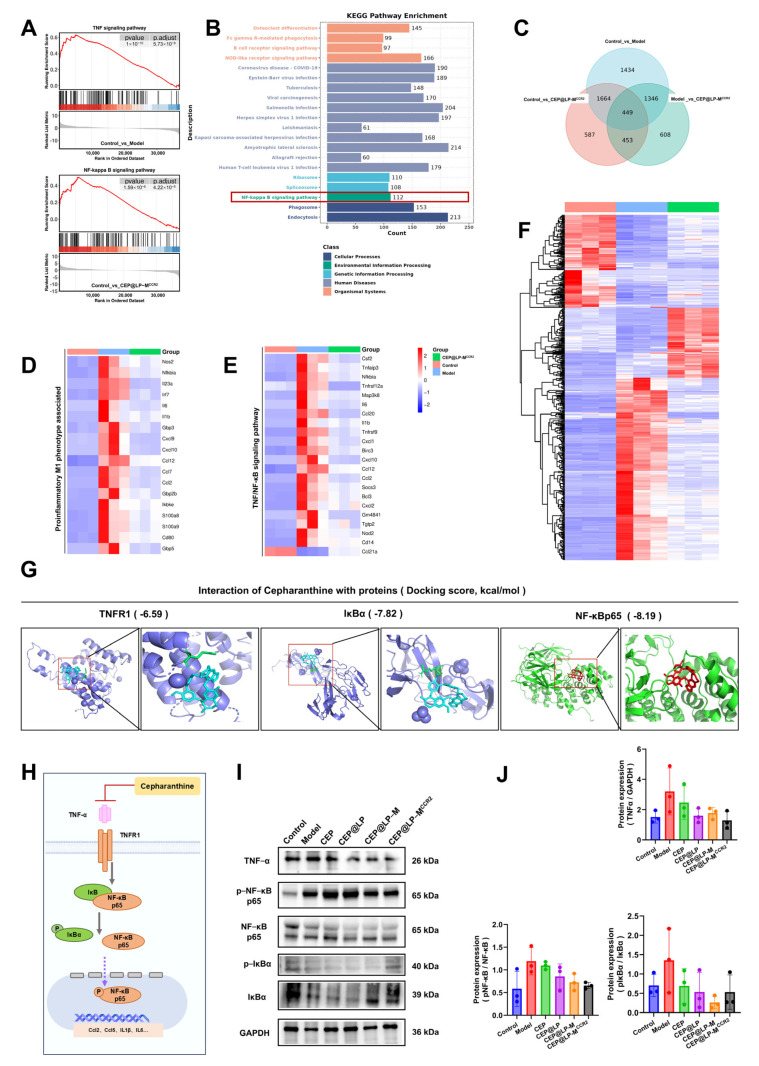
CEP@LP-M^CCR2^’s overall effects on treating ALI by RNA-seq analysis of lung tissues. (**A**) GSEA in Control vs. Model, Control vs. CEP@LP-M^CCR2^. (**B**) KEGG pathway enrichment in the group cluster of Model, CEP@LP-M^CCR2^ and Control. (**C**) DEGs from different comparison groups intersected in a Venn diagram, including Control vs. Model, Model vs. CEP@LP-M^CCR2^, Control vs. CEP@LP-M^CCR2^. (**D**) Cluster analysis of M1 macrophage-associated DEGs among all groups. (**E**) Cluster analysis of the key DEGs in the TNF/NF-κB signaling pathway. (**F**) Cluster analysis of a total of 449 intersected DEGs in A by heatmap. (**G**) Binding activity of CEP to TNFR1, IκBα, and NF-κB p65 proteins showed by an in silico molecular docking analysis. (**H**) The pathway of CEP@LP-M^CCR2^ may influence the progression of ALI through the TNF/NF-κB signaling axis. (**I**) Protein expressions of key members in the TNF/NF-κB pathway by Western blot, including TNF-α, phosphorylated (p-NF-κB p65) and NF-κB p65, p-IκBα, IκBα. (**J**) Normalized expression of the proteins calculated by Image J. Internal control, GAPDH. Signal pathways of particular interest are highlighted with red boxes.

We initially performed GSEA for the comparisons of Control vs. Model and Control vs. CEP@LP-M^CCR2^. The results revealed a trend toward enrichment in the NF-κB signaling pathway and TNF signaling pathway in these comparisons (Figure 8A). This observation was further supported by the KEGG pathway enrichment results from the clustered samples of the Control, Model, and CEP@LP-M^CCR2^ groups (Figure 8B). Next, we intersected the differentially expressed genes (DEGs) between the ALI model group and control group, CEP@LP-M^CCR2^ group and control group, and model group and CEP@LP-M^CCR2^ group in a Venn diagram, revealing 449 shared DEGs (Figure 8C). All shared DEGs were aggregated into a heatmap, indicating that mRNA levels in control group and CEP@LP-M^CCR2^-treated group were completely distinct from those in the model group (Figure 8F). Compared to the control group, some mRNA levels in theCEP@LP-M^CCR2^-treated group were relatively low, yet opposite to those in the model group, suggesting recovery from the inflammatory lung state. Furthermore, approximately half of the DEGs were upregulated in the model but downregulated after CEP@LP-M^CCR2^ treatment, indicating their association with the ALI.

Subsequent analysis of these 449 genes, cluster analysis of key members of the TNF/NF-κB signaling pathway revealed downregulated expression levels in the control group and the CEP@LP-M^CCR2^ group, while the model group exhibited upregulated expression (Figure 8E). RNA-seq results revealing that the model group exhibited upregulation of 22 genes within the TNF signaling pathway, including *clAP* (*Birc3*), *A20* (*Tnfaip3*), chemokines involved in leukocyte recruitment (e.g., CCL2, CCL20, CXCL1-3), and inflammatory cytokines (e.g., IL1β, IL6), whereas the CEP@LP-M^CCR2^ -treated group downregulated these genes (Figure 8E). This indicates that CEP@LP-M^CCR2^ inhibits the activation of this pathway. In addition, through heatmap analysis, we observed several genes associated with macrophage M1 polarization exhibiting lower expression levels in the control group and the CEP@LP-M^CCR2^ group compared to the model group (Figure 8D). Our experimental results mentioned earlier also validated these RNA sequencing findings.

In summary, CEP@LP-M^CCR2^ maintains macrophage polarization by regulating multiple pro-inflammatory signaling pathways, demonstrating potential therapeutic effects for various inflammatory diseases, including ALI, consistent with experimental findings.

### 3.10. Potential Pathways of CEP@LP-M^CCR2^ in ALI Mice

Based on RNA-seq results, the TNF/NF-κB signaling pathway was selected as a candidate pathway to explore the potential mechanism underlying the therapeutic effects of CEP@LP-M^CCR2^ on ALI. This pathway is widely recognized as a classic pro-inflammatory signaling pathway involved in inflammatory lung diseases progression [46,55,56]. First, we conducted computational molecular docking analysis to investigate potential interactions between CEP and key members of this pathway. Analysis revealed strong binding activity between CEP and TNFR1, IκBα and NF-κB p65 (Figure 8G).

In the progression of ALI, LPS induces the release of pulmonary TNF-α, a key mediator that induces apoptosis and enhances immune responses; TNF-α activates TNF receptor 1 (TNFR1) and initiates the classic TNF/NF-κB pathway (Figure 8H). Western blot analysis was performed to further validate the lung tissue findings. Compared with the control group, the phosphorylation of IκBα and p65 were upregulated in the ALI model group; however, CEP@LP-M^CCR2^ treatment reversed these protein upregulations, indicating inhibition of TNF/NF-κB signaling axis activation. The results indicate that CEP@LP-M^CCR2^ suppresses the activation of the TNF/NF-κB pathway, which may represent a potential mechanism underpinning its therapeutic effects against ALI (Figure 8I,J).

This mechanism involves regulating macrophage polarization and repairing the pulmonary barrier. It has been reported that activation of the TNF/NF-κB pathway, along with dysregulation of macrophage polarization, are factors that exacerbate ALI and lung barrier injury, further supporting our findings [55].

## 4. Discussion

In recent years, the development of nanomaterials has brought some changes to the medical field [57], including in research on lung diseases. Cell membrane-coated liposomes (LP-M) combine the advantages of artificially synthesized nanoparticles and natural cell membranes and have the advantages of enhanced biocompatibility, prolonged circulation time and improved targeting [58]. Among cell membranes, most of these nanodelivery systems are applied to target lesion sites through homogeneous targeting mechanisms. At present, there is still a lack of ALI treatment strategies that use HEK293T cell membranes as coatings. Theoretically, HEK293T cells offer advantages such as a rapid cell cycle, high transfection efficiency, and a robust capacity for expressing diverse target proteins. Our results indicate that compared with CEP@LP, CEP@LP-M could enhance biocompatibility to a greater degree and demonstrates superior therapeutic efficacy over CEP (Table S3). Consequently, membranes derived from HEK293T cells could be leveraged to develop possibilities for the treatment of ALI.

Due to the differences in pathological environments, customizing characteristic biofilms through biological modification may be an important research topic for LP. Advances in cell separation techniques and engineering methods have promoted the development of more complex cell membrane coating nanomaterials [59]. These advancements allow for the introduction of additional factors with specific functions on the surface of nanoparticles, such as specific ligands, antibodies or imaging agents. This further enhances their targeting accuracy, therapeutic effect and diagnostic ability. We constructed bioengineered cell membranes overexpressing CCR2 and achieved inflammation targeting in vivo. Though some circulating macrophages express CCR2, our strategy has merits: engineered cell systems show better reproducibility than primary cells. Furthermore, compared with the hybrid membrane strategy of platelets and CCR2-overexpressing cells [60], we established stably CCR2-overexpressing HEK293T cells through lentiviral transfection. This approach elevates target protein expression, allowing for precise regulation of surface CCR2 density, which avoids targeting efficiency fluctuations from uneven native membrane protein expression and reduces immune clearance risk induced by these proteins. Another point is that primary cells are more sensitive and variable. For instance, macrophages have two phenotypes, M1 type and M2 type. Meanwhile, the two phenotypes can transform into each other under certain conditions. Macrophages in M1 state can promote the progression of diseases, while cells in M2 state have anti-inflammatory effects. However, it is difficult to distinguish and retain beneficial cell membranes in vitro, which makes this strategy challenging.

In addition, the cell membrane of the CCR2-293T cell line we constructed and extracted not only achieved targeting but also played a blocking role. RNA-seq results of CEP@LP-M^CCR2^ showed that this nanoparticle not only reduced the expression of CCL2 but also decreased the expression of other chemokines and cytokines. Additionally, we employed further experiments to support CCR2–CCL2. In vivo, we prepared a drug-free CCR2 cell membrane solution (CCR2CM) and administered it in parallel to disease mouse models to establish a parallel control. Compared with the Model group, both the CCR2CM and CEP@LP-M^CCR2^ groups exhibited reduced FPKM values for CCL2 (Figure S1A), indicating that both CCR2CM and CEP@LP-M^CCR2^ can downregulate CCL2 expression at the transcriptional level. This conclusion was further supported by qPCR validation experiments (Figure S1B). Compared with the CEP@LP-M^CCR2^ group, CCL2 expression levels were lower in the CCR2CM group (Figure S1B,C), but the results are modest. However, given that the sole difference between CEP@LP-M^CCR2^ and CCR2CM lies in whether CEP@LP is loaded, we hypothesize that the downregulation of CCL2 expression results from the combined effects of CEP@LP and CCR2CM, and that CCR2CM alone can reduce CCL2 expression in the lungs of diseased mice. This effect may be attributed to negative feedback regulation triggered by the binding of CCR2 (on the surface of CCR2CM) to CCL2 [61]. In vitro, compared with the Control group, CCL2 levels increased in the Model group, and the cellular uptake efficiency of Did@LP-M^CCR2^ was enhanced due to the binding of CCR2 on the cell membrane to CCL2. Conversely, compared with the Model group, Bindarit inhibited LPS-induced endogenous CCL2 secretion in the Experimental group, resulting in a reduction in the cellular uptake of Did@LP-M^CCR2^ (Figure S2A,B). In summary, these results could support CCR2-CCL2-mediated targeting.

Despite the substantial progress made, challenges still exist. The scalability and repeatability of the cell membrane coating method need to be improved. It should be noted that a comprehensive assessment of the long-term safety and potential immunogenicity of these nanomaterials must be conducted to determine whether the original cell membrane will be damaged during the preparation process. Furthermore, the use of a single cell membrane coating in our current research still has certain limitations.

In addition to the nanocarriers, the components of the encapsulation are also very important. The first-line drugs currently used for the treatment of ALI mainly include glucocorticoids such as dexamethasone and methylprednisolone. These drugs have shown certain efficacy in delaying the progression of the disease and improving lung function. However, oral administration often leads to adverse reactions in the gastrointestinal tract and the liver. Furthermore, due to the lack of organ and cell specificity, the therapeutic effects of these drugs are severely limited. Our next research step will focus on enhancing the encapsulation components. Although we loaded the CEP derived from Chinese herbal medicine into lipid vesicles coated with cell membranes for multi-targeted treatment against ALI, the therapeutic effect of single-drug treatment still has its limitations. For instance, our research has found that CEP has certain potential in restoring the endothelial barrier, but if multiple drugs with different mechanisms of action are used in synergy, it may be more helpful in addressing the multifaceted nature of ALI pathology. For instance, one drug might target the alleviation of the accumulation of inflammatory factors, while another drug might focus on promoting the recovery of the diffuse damage to the epithelium and endothelium. Compatibility with appropriate carriers can enhance the therapeutic effect of drugs.

In conclusion, we prepared drug-loaded cell membrane liposomes, achieving potential targeting and attenuation of ALI. This original nanomedicine can improve bioavailability, alleviate ALI, relieve inflammation and promote the recovery of the lung barrier.

## 5. Conclusions

In this study, we innovatively designed CEP@LP-M^CCR2^. Our research mainly focuses on two goals. Firstly, we demonstrated that this new type of nanocarrier has the potential to target inflammatory lesions in the lungs through the CCR2-CCL2 binding mechanism. Subsequently, the results of in vitro and in vivo experiments demonstrated the therapeutic potential of CEP@LP-M^CCR2^ in the treatment of ALI, including anti-inflammatory action, reduction in the magnitude of the inflammatory response, suppression of M1 macrophage polarization, preservation of epithelial integrity, and reductions in pulmonary edema and tissue injury. Moreover, RNA sequencing indicates that CEP@LP-M^CCR2^ may alleviate ALI by targeting the TNF/NF-κB signaling axis.

## Data Availability

The data presented in this study are openly available from the National Genomics Data Center under the accession number CRA035944.

## Supplementary Materials

The following supporting information can be downloaded at: https://www.mdpi.com/article/10.3390/cells15030292/s1. Table S1: Information related to the CCR2 plasmid; Table S2: qPCR Primer Information; Table S3: The normalized relative mRNA expression levels in different groups. Figure S1: Transcriptome Data Analysis of the *Ccl2* Gene; Figure S2: Targeted uptake in vitro by using flow cytometry; Figure S3: Construction and characterization of CEP@LP.

## Abbreviations

The following abbreviations are used in this manuscript:

HEK293T	Human Embryonic Kidney 293T Cells
MLE-12	Mouse Lung Epithelial-12 Cells
RAW264.7	Murine Leukemic Monocyte-Macrophage 264.7 Cells
BEAS-2B	Bronchial Epithelial Cells
CEP	Cepharanthine
LP	Liposome
CEP@LP	CEP Liposomes
CEP@LP-M	Empty vector-transfected Cell Membrane-camouflaged CEP Liposomes
CEP@LP-M^CCR2^	CCR2-expressing Cell Membrane-camouflaged CEP Liposomes
ALI	Acute Lung Injury
EV	Empty Vector
