# Supplementary material for: Engineered CCR2 Cell Membrane-Wrapped Cepharanthine Liposomes for Potential Targeted Attenuation of Acute Lung Injury
Source: Cells. 2026 Feb 4;15(3):292. doi: 10.3390/cells15030292 (PMC12896890; doi:10.3390/cells15030292)
Supplement: Supplementary file 1 [file cells-15-00292-s001.zip › cells-4121640-supplementary.pdf]

Original article, Supporting Information

Engineered CCR2 Cell Membrane-Wrapped Cepharanthine Liposomes for Potential Targeted Attenuation of Acute Lung Injury

Yifan Qing [1,2], Wenbo Zhao [2,3], Liangliang Xue [1,2], Yu Luo [2], Yuhao Gao [3], Xiang Sun [2,3], Fan Li [1,2], Linxuan Dai [5], Jing Mo [2], Guoqing Xu [2], Zenghao Bi [2,3], Suleixin Yang [2,4], Woo Tiam Hee [6], Jie Li [2,4,*] and Liang Leng [1,2,*]

[1] School of Basic Medical Sciences, Chengdu University of Traditional Chinese Medicine, Chengdu 611137, China;

[2] Institute of Herbgenomics, Chengdu University of Traditional Chinese Medicine, Chengdu 611137, China;

[3] College of Pharmacy, Chengdu University of Traditional Chinese Medicine, Chengdu 611137, China;

[4] Innovative Institute of Chinese Medicine and Pharmacy, Chengdu University of Traditional Chinese Medicine, Chengdu 611137, China;

[5] School of Medical and Life Sciences, Chengdu University of Traditional Chinese Medicine, Chengdu 611137, China;

[6] M. Kandiah Faculty of Medicine and Health Sciences, Universiti Tunku Abdul Rahman, Selangor 43000, Malaysia.

* Correspondence: Jie Li; Liang Leng

Supporting Information

Table S1. Information related to the CCR2 plasmid.

Gene	CCR2	Gene ID	729230
Species	Human	Size of the gene	9385bp
Resistance	Amp	Restriction site	EcoRI、BamHI
Vector	pCDH-GFP+Puro-3xFlag		
Plasmid	pCDH-CCR2-GFP+Puro-3xFlag		
Forward primer	CCR2-F GATTCTAGAGCTAGCGAATTCGCCACCATGCTTTCTACGTCTC		
Reverse primer	CCR2-R AACGTTCTGTTCCTTCCACGTCCTAGGCTGATGTTTCTGGTA		

Table S2. qPCR Primer information. (F: Forward, R: Reverse, H: Human, M: mouse)

Primer name	Sequence	Primer name	Sequence
H-CCR2-F	CAACGAGAGCGGTGAAGAAGTC	H-CCR2-R	GAGTAGAGCGGAGGCAGGAG
H-GAPDH-F	GTGGACCTGACCTGCCGTCTAG	H-GAPDH-R	GAGTGGGTGTCGCTGTTGAAGTC
H-TNFα-F	ATGAGCACTGAAAGCATGATCCG	H-TNFα-R	AGGAGAAGAGGCTGAGGAACAAG
H-IL6-F	AGAGTAGTGAGGAACAAGCCAGAG	H-IL6-R	GGCATTTGTGGTTGGGTCAGG
H-IL1β-F	GCACCTGTACGATCACTGAACTG	H-IL1β-R	CACTTGTTGCTCCATATCCTGTCC
H-CCL2-F	CCAGCAGCAAGTGTCCCAAAG	H-CCL2-R	TGCTTGTCCAGGTGGTCCATG
M-GAPDH-F	AGAAGGTGGTGAAGCAGGCATC	M-GAPDH-R	CGAAGGTGGAAGAGTGGGAGTTG
M-TNFα-F	GTGCCTATGTCTCAGCCTCTTCTC	M-TNFα-R	TGGTTTGTGAGTGTGAGGGTCTG
M-IL6-F	GACTTCCATCCAGTTGCCTTCTTG	M-IL6-R	GACAGGTCTGTTGGGAGTGGTATC
M-IL1β-F	CTCGCAGCAGCACATCAACAAG	M-IL1β-R	CCACGGGAAAGACACAGGTAGC
M-CCL2-F	ACTCACCTGCTGCTACTCATTCAC	M-CCL2-R	TTCTTTGGGACACCTGCTGCTG
M-iNOS-F	CTATGGCCGCTTTGATGTGC	M-iNOS-R	TTGGGATGCTCCATGGTCAC
M-Arg1-F	ACATTGGCTTGCGAGACGTA	M-Arg1-R	ATCACCTTGCCAATCCCCAG
M-CD86-F	ATCTGCCGTGCCCATTTACA	M-CD86-R	CAACTTTTGCTGGTCCTGCC
M-CD163-F	GAGAAGACGCTGGTGTGACA	M-CD163-R	CCAAGCTGTCTGCAAACCAC
M-CD206-F	AAATGGCTTCCTGGAGAGCC	M-CD206-R	ACCCTCCGGTACTACAGCAT
M-CD80-F	GGCCCGAGTATAAGAACCGG	M-CD80-R	GTATGTGCCCCGGTCTGAAA
M-cadherin1-F	TTCTGATCCTGCTGCTCCTACTG	M-cadherin1-R	TCTTCTTCTCCACCTCCTTCTTCATC
M-occludin-F	CTGACCTTGAGTGTGGATGACTTC	M-occludin-R	CCTCTTGCCCTTTCCTGCTTTC

Table S3. The normalized relative mRNA expression levels in different groups

		Control	Model	CEP	CEP@LP	CEP@LP-M	CEP@LP-M^{CCR2}
BEAS-2B	TNF-α	2.624±1.512	14.9±2.841	0.01±0.001	0.018±0.006	0.006±0.005	0.014±0.01
	IL-1β	0.873±0.157	81.056±3.376	38.026±2.636	10.89±1.719 [a]	6.582±0.682 [a]	6.753±0.446 [a]
	IL6	0.916±0.133	12.133±0.504	9.351±1.611	8.857±0.154	7.311±1.534	6.497±1.174 [a]
MLE-12	TNF-α	1.004±0.104	4.801±1.242	2.008±0.145	2.385±0.097	1.371±0.136	0.939±0.068 [b]
	IL-1β	0.984±0.499	2.385±0.584	0.89±0.216	0.211±0.05	0.421±0.092	0.286±0.12
	IL6	0.989±0.215	2.533±0.111	1.203±0.167	0.657±0.246	1.313±0.31[b]	1.084±0.299
RAW264.7	Arg1	1.043±0.04	0.172±0.009	0.825±0.176	2.092±0.111 [a]	0.273±0.021 [a,b]	0.693±0.034 [b,c]
	iNOS	1.009±0.306	214.336±25.91	0.604±0.228	0.325±0.16	0.25±0.183	0.087±0.011
	CD86	1.16±0.14	2.36±0.148	0.645±0.064	0.92±0.017 [a]	1.215±0.047 [a,b]	0.462±0.034 [b,c]
	CD163	1.059±0.236	0.942±0.288	1.333±0.129	0.177±0.036 [a]	0.219±0.057 [a]	0.259±0.049 [a]
	CD206	0.998±0.043	0.462±0.012	1.202±0.145	0.756±0.111 [a]	0.892±0.056	1.055±0.278
Lung	E-cadherin	1.09±0.1	0.364±0.009	0.796±0.042	0.651±0.014 [a]	0.542±0.021 [a]	0.715±0.044 [c]
	Occludin	1.09±0.111	0.474±0.008	0.82±0.032	0.523±0.019 [a]	0.737±0.077	0.632±0.168
	TNF-α	0.847±0.17	1.733±0.285	1.081±0.239	0.781±0.028	0.249±0.032 [a,b]	0.274±0.068 [a,b]
	IL-1β	1.171±0.203	254.903±7.98	50.474±2.029	44.093±3.124	24.763±4.496 [a,b]	26.804±1.253 [a,b]
	IL6	0.883±0.295	3.564±0.051	1.434±0.121	0.414±0.02 [a]	0.262±0.038 [a]	0.152±0.039 [a]
	CCL2	0.863±0.208	21.059±1.681	2.369±0.097	2.258±0.127	1.226±0.242	0.545±0.109 [a]
	Arg1	0.913±0.098	0.666±0.073	2.248±0.492	1.441±0.061 [a]	2.708±0.075 [b]	2.278±0.104 [b]
	iNOS	1.134±0.573	6.246±0.788	1.242±0.682	1.309±0.642	1.48±0.468	0.427±0.319
	CD86	1.227±0.404	11.095±0.674	3.334±0.299	4.846±0.57 [a]	3.008±0.207 [b]	2.512±0.158 [b]
	CD163	0.914±0.085	0.454±0.098	0.624±0.042	0.589±0.032	0.747±0.067	0.713±0.005
	CD80	1.025±0.231	4.872±0.754	4.361±2.055	3.78±0.107	2.379±0.281	2.563±0.311

Statistical Analysis: a, $p < 0.05$ vs CEP; b, $p < 0.05$ vs CEP@LP; c, $p < 0.05$ vs CEP@LP-M

Note: All data are presented as Mean ± SD. The statistical analysis in this table applies only to different liposome groups.

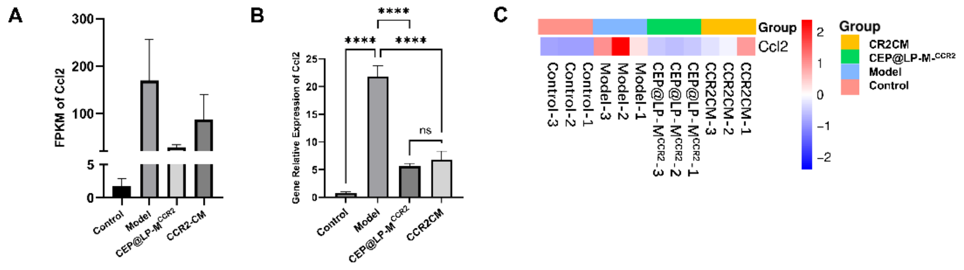

Figure S1. Transcriptome Data Analysis of the *Ccl2* Gene. (A) Fragments Per Kilobase of exon model per Million mapped fragments (FPKM) of *Ccl2*. (B) The mRNA expression of *Ccl2* by qRT-PCR. (C) Heat map of the expression of *Ccl2* in different groups.

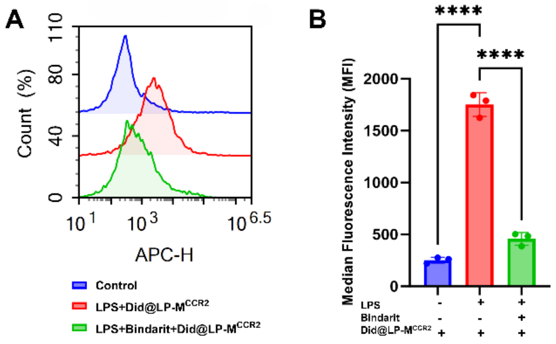

Figure S2. (A-B) Targeted uptake *in vitro* by using flow cytometry. Targeted to BEAS-2B. (Bindarit is a selective inhibitor of CCL2.)

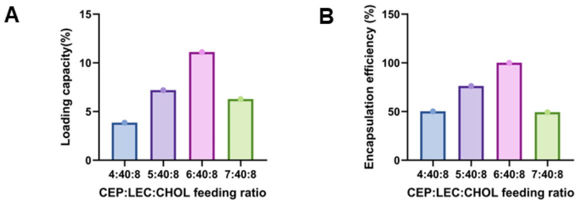

Figure S3. Construction and characterization of CEP@LP. (A) The loading capacity of CEP in LP at different feeding ratios. (B) The encapsulation efficiency of CEP in LP at different feeding ratios.
